# Analysis of bigmouth buffalo *Ictiobus cyprinellus* spawning phenology in Minnesota reveals 50-year recruitment failure and conservation concern

**DOI:** 10.1038/s41598-024-70237-5

**Published:** 2024-09-03

**Authors:** Alec R. Lackmann, Sam Seybold, Ewelina S. Bielak-Lackmann, Walt Ford, Malcolm G. Butler, Mark E. Clark

**Affiliations:** 1 Department of Mathematics and Statistics, University of Minnesota Duluth, 140 Solon Campus Center, 1117 University Drive, Duluth, MN 55812 USA; 2 Department of Biology, University of Minnesota Duluth, 1035 Kirby Drive, SSB 207, Duluth, MN 55812 USA; 3 Aitkin County Soil and Water Conservation District, 307 2nd St NW #216, Aitkin, MN 56431 USA; 4 United States Fish and Wildlife Service, Rice Lake National Wildlife Refuge, 36289 State Hwy. 65, McGregor, MN 55760 USA; 5 Department of Biological Sciences, North Dakota State University, Dept. 2715, PO Box 6050, Fargo, ND 58108 USA

**Keywords:** Ecology, Conservation biology, Zoology, Ichthyology, Freshwater ecology

## Abstract

The bigmouth buffalo *Ictiobus cyprinellus* (Catostomidae) is a freshwater fish native to North America that is known for its longevity. During the 1970s, the bigmouth buffalo was recorded as declining in Canada, Minnesota, and North Dakota and became a protected species in Canada. In the USA, population declines are exacerbated by wasteful recreational bowfishing, lack of fisheries management, and overall lack of knowledge. However, recent studies have revealed the exceptional lifespan of bigmouth buffalo, their negligible senescence, slow growth, delayed maturity, and episodic recruitment. Yet little is known about the spawning phenology of bigmouth buffalo, nor their age demographics in east central Minnesota. In this 2021–2023 study of bigmouth buffalo from Rice Lake National Wildlife Refuge we found that 99.7% (389 of 390) of the extant population hatched prior to 1972 despite annual spawning in Rice Lake. Moreover, recruitment success declined significantly since water control measures were established (1953). We found males arrive to spawning grounds with females but depart later, that both the midpoint and duration of spawn significantly vary across years, and that more massive females of the same age range invest disproportionately more in ovaries. Extensive post-spawn seining revealed bigmouth buffalo young-of-the-year in low numbers, but by mid-to-late summer they were no longer evident having likely succumbed to predation. Overall, these findings thoroughly reveal one of the oldest populations of vertebrate currently known (median age of 79 years as of 2024) and expose the stark vulnerability of a bigmouth buffalo population for which substantial recruitment has not occurred for more than six decades. Multiple lines of evidence indicate that the long-lived bigmouth buffalo is vulnerable, that a precautionary approach is immediately needed, and that the unlimited and unregulated kill-fishery be closed.

## Introduction

The bigmouth buffalo *Ictiobus cyprinellus* is a unique freshwater fish native to North America that is of conservation concern on multiple levels. It is the only member of the Catostomidae that tends to filter-feed in the open water on plankton^1^, and it can reach a larger size (in body mass) than any other catostomid^2^. Native to the Mississippi and Hudson Bay (southern extent) drainages, it is a geographically widespread species whose endemic range has contracted in the north, including Canada, North Dakota, and Minnesota^2^. Indeed, careful monitoring of bigmouth buffalo in Canada during the mid twenty-first century led to concerns that populations were declining because of commercial fishing, habitat fragmentation, and competition with invasive carp^3^. Significant population declines have now become evident in multiple regions^4^. A recent study of bigmouth buffalo in North Dakota revealed population declines^5^, while studies from Minnesota and Arizona have shown a similarly long-lived life history that is vulnerable to overexploitation^6,7^. Moreover, major conservation assessments for bigmouth buffalo remain outdated, with the most recent assessment from the International Union for Conservation of Nature occurring in 2012^8^ and from US-based NatureServe in 2015^9^. These conservation evaluations were based on earlier life history data for bigmouth buffalo that underestimated age at maturity and lifespan by an order of magnitude^4–7^, and before recognition of the threat posed by the recent exponential increase in recreational bowfishing, a new exploitation with unprecedented waste and virtually no regulatory oversight^10–12^.

Systemic misconceptions around bigmouth buffalo ecology and management stem from long-held biases. Long labeled as “rough fish”^6,13^, buffalofishes and other catostomids have been subject to unfounded biases that fisheries scientists have warned against for more than a century^13–17^. Such biases continue within fisheries management. For example, the derogatory label “rough fish”^13^ persists in many current fishing regulations^18–20^, even as lethal sport fisheries have emerged that target and remove buffalofishes as game fish in large numbers^5,6,10,12,13^. The “rough fish” label connotes lack of ecological value to anglers as well as to the public^13^. As a result these species are understudied, research on them is underfunded, and their fisheries are typically unregulated and unmonitored^17,21^. The following examples illustrate this point: (1) native “rough fish” receive 11-fold less study than traditional “game” fish^13^; (2) unlimited take still exists on bigmouth buffalo across the majority of their US range (including ND and MN), yet Canada established no-take protections during the 1980s on contiguous waters where centenarian populations were more recently discovered^6^; (3) since 2010 recreational sport bowfishing exhibited exponential growth in participation and levels of exploitation that can exceed commercial harvests, yet management of bowfisheries remains almost non-existent^6,10–12,17,22–24^; and (4) many recent studies on these species lacked grant funding^7,22–24^.

Bigmouth buffalo exhibit a complex life history that was long overlooked. Previously thought to be a fast-growing fish that typically lived 10–20 years maximum^1,25^, bigmouth buffalo are now known as one of the longest-lived vertebrates, capable of living more than 125 years^4,6^. Bigmouth buffalo exhibit a suite of traits such as slow asymptotic growth, delayed maturity, and iteroparity, life history features associated with seasonal environments^26^, and with residency in habitats where periods of successful recruitment may be separated by significant time intervals^4–7^. Specifically, bigmouth buffalo reproductive biology is characterized by migrating long distances to flooded vegetation for spawning^27^, low investment per egg, and provision of no parental care^1^. Thus, when spawning does occur, eggs and fry are vulnerable to predation as well as fluctuating water levels in nursery habitat^4^. Bigmouth buffalo exhibit negligible senescence in physiological systems as they approach 100 years old^28^, another dimension of a slow life history. Nevertheless, little is known about the migration and spawning phenology of this long-lived species, its spawning success, nor the population demographics of bigmouth buffalo in east central Minnesota; information that is necessary for sustainable fisheries management of this exceptional freshwater fish.

In this study we investigate the spawning phenology and population demographics of bigmouth buffalo from Rice Lake National Wildlife Refuge and the Rice River watershed of east-central Minnesota. We quantify sex-specific spawning migration timing and spawning morphologies. We investigate age demographics based on annuli in thin-sectioned otoliths and assess spawning success via sampling for young-of-the-year post spawn. Using this information, we evaluate recruitment patterns of bigmouth buffalo over the past century.

## Methods

### Study site

**Figure 1:**
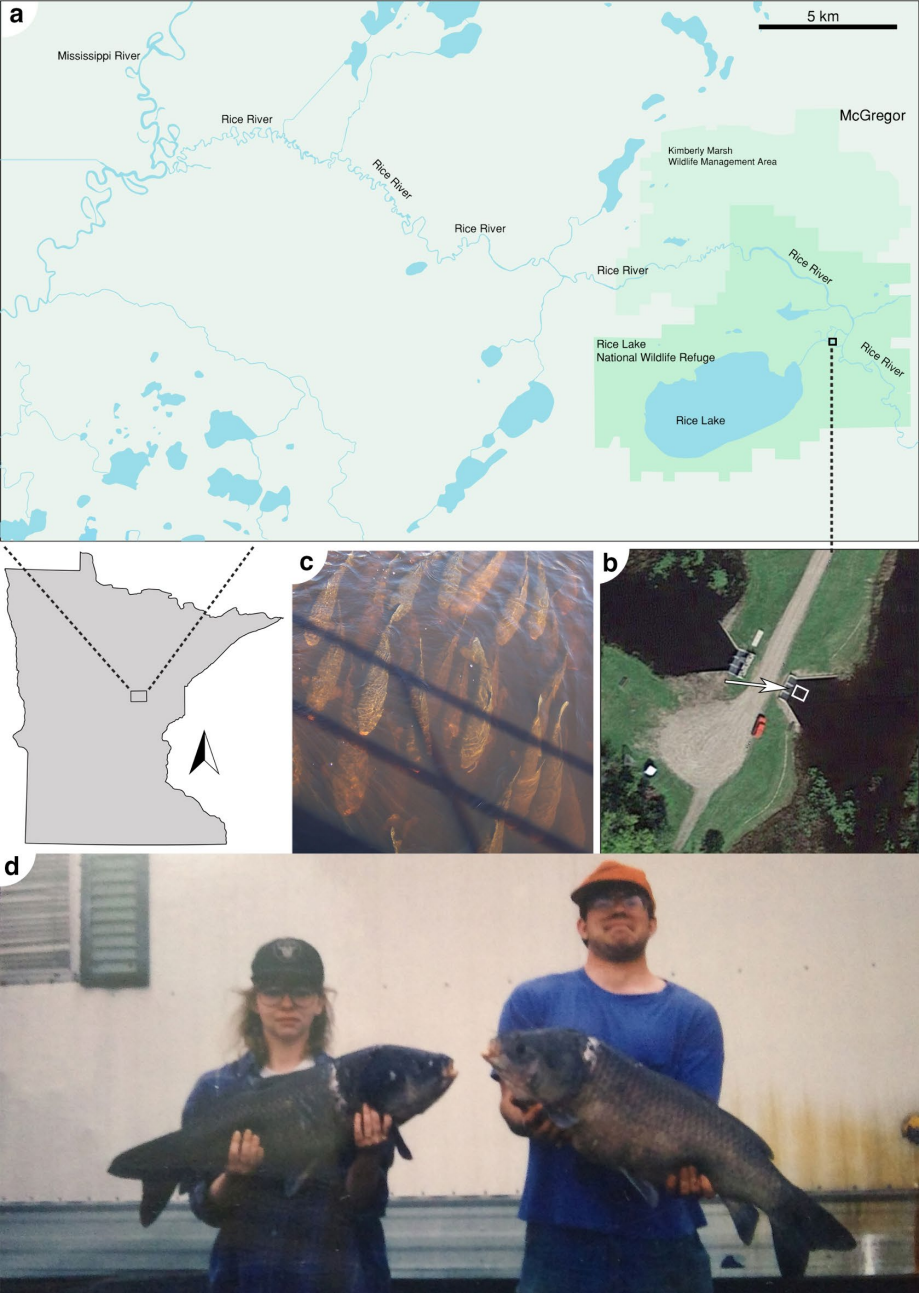
Rice River and Rice Lake National Wildlife Refuge (RLNWR), Minnesota. (**a**) Detailed view of RLNWR, the Rice River (a 92 km tributary of the Mississippi River), and the bigmouth buffalo *Ictiobus cyprinellus* spawning site of Rice Lake in east central Minnesota. (**b**) Aerial image of the lakeside water control structure (see “Methods”), located 1.7 river km downstream of Rice Lake, where the channel is < 6 m wide. (**c**) Downstream image from the lakeside water control structure showing aggregations of bigmouth buffalo moving upstream on 6 May 2013. Image (**c**) is approximated by the white box in b in the direction indicated by the arrow. (**d**) Two large (> 9 kg) bigmouth buffalo harvested during May 1994 by spearing from the lakeside water control structure. The bigmouth buffalo were harvested while swimming upstream towards Rice Lake on their way to spawn. We retrieved map information from Google Maps (retrieved February 2024) and organized its layout using Adobe Inc. software including Adobe Illustrator (Creative Cloud version; https://www.adobe.com).

The Rice River and Rice Lake complex is a river-marsh ecosystem with interspersed woodland, with reproducing populations of bigmouth buffalo and other native fishes. The Rice River is a 92 km tributary of the Mississippi River that originates in the Solana State Forest approximately 11 km south of Rice Lake National Wildlife Refuge (RLNWR), operated by the United States Fish and Wildlife Service (USFWS). RLNWR is located 8 km south of McGregor, Minnesota (Fig. 1a), and the Rice River passes within 2 km of the 14.6 km^2^, < 2 m deep Rice Lake, and the Rice River hydrologically connects to Rice Lake via its own tributaries (Fig. 1a,b). The Rice River eventually drains into the Mississippi River approximately 32 km west of RLNWR (Fig. 1a)^29^. Across centuries, bigmouth buffalo have been observed during spring migrating upstream along the Rice River to spawn in flooded vegetation such as wild rice *Zizania palustris* in Rice Lake^29,30^, an ecosystem known for its vast stands of wild rice and millions of migratory waterbirds^29–31^. In addition, northern pike *Esox lucius*, white sucker *Catostomus commersonii*, yellow perch *Perca flavescens*, bullhead *Ameiurus* spp., various Centrarchidae, and numerous other native fishes spawn in Rice Lake^29,31^.

Bigmouth buffalo migrating in Rice River are visible from a few points along the river channel, and they have been exploited from the upstream “lakeside” water control structure for decades (Fig. 1b–d). This is the narrowest point (< 5 m across) of the river channel (Fig. 1b), and is one of two control structures along the river used to regulate water levels for wild rice production^29,32^. The lakeside water control structure is 1.7 river kilometers (rkm) from Rice Lake and was the point of observation and data collection for this study because it is where anglers congregate. Another water control structure is located 8 km downstream, but anglers do not exploit bigmouth buffalo from this location because the river channel is too wide (16 m across). Both water control structures were constructed during 1950–1953. First, a 1.22 m stoplog was installed as the lakeside structure, whereas two 3.66 m gates were installed for the downstream structure^33^. In 1979, both structures were updated such that the lakeside structure now consists of three 1.52 m concrete-walled channels with operational iron gates, and the downstream structure consists of four 3.66 m gates and a 1.22 m stoplog slot^29,32,33^.

### Monitoring the bigmouth buffalo migration

We monitored bigmouth buffalo spawning migration at the lakeside water control structure in Rice Lake National Wildlife Refuge following spring ice out in each of years 2021–2023 to quantify spawning phenology.

In 2021, we focused monitoring on bigmouth buffalo captured by any means (bowfishers, spearfishers, or traditional anglers) by recording the date, time, and direction (i.e., upstream, downstream) of movement of any bigmouth buffalo captured. Duration and intensity of our monitoring varied according to fish sightings, and RLNWR is closed between sunset and sunrise and thus all monitoring took place during the day across all years. For our initial visit on 8 April 2021 during which many area lakes were still frozen, we monitored the reach for approximately two hours during daylight hours and returned in 13 days to monitor the reach again for the same amount of time (Table 1). We then returned after 3 days and again monitored the water control structure for two hours (Table 1). Once we made the first bigmouth buffalo sighting we then monitored the water control structure at least two hours daily. Once we had observed more than six bigmouth buffalo in a 2-h period, we increased our monitoring to approximately 12 h daily (from approximately 8:00 am to 8:00 pm) and returned to the water control structure for such monitoring until subsequent days in which no bigmouth buffalo was observed during the 12-h period (Table 1). At this point we discontinued monitoring and determined the spawning period to have ended.

**Table 1:** Capture data by date with observation notes for bigmouth buffalo (BMB) collected from Rice Lake National Wildlife Refuge in 2021–2023.

Date	Year	#BMB	#F	#M	#PSF	BMB/h	MD	Note
8 April	2021					0		No BMB observed
21 April	2021					0		No BMB observed
24 April	2021					0		No BMB observed
25 April	2021					3	1	
26 April	2021					1	2	
27 April	2021					0	3	No BMB observed
28 April	2021					0	4	No BMB observed
29 April	2021					0	5	No BMB observed
30 April	2021	4	3	1		6	6	
1 May	2021	6	4	2			7	
2 May	2021	8	7	1			8	
3 May	2021	1	1				9	Releasing eggs out of water
4 May	2021	4	4		3		10	
5 May	2021	4	4		4		11	
6 May	2021	1	1		1		12	
7 May	2021	2		2			13	
8 May	2021	7	1	6	1		14	
9 May	2021	3		3			15	
10 May	2021					0		No BMB observed
11 May	2021					0		No BMB observed
Total	2021	40	25	15	9			
20 April	2022					0		No BMB observed
21 April	2022					0		No BMB observed
22 April	2022					0		No BMB observed
23 April	2022					0		No BMB observed
24 April	2022					0		No BMB observed
25 April	2022					0		No BMB observed
26 April	2022					0		No BMB observed
27 April	2022					0		No BMB observed
28 April	2022	4		4		60	1	Bowfishers discarded catch
29 April	2022					100	2	90% of BMB observed were probable males
30 April	2022	6	2	4			3	
1 May	2022	7	2	5			4	
2 May	2022	4	3	1			5	
3 May	2022	12	9	3			6	
4 May	2022	21	11	10			7	
5 May	2022	19	13	6			8	
6 May	2022	22	14	8			9	
7 May	2022	20	13	7			10	
8 May	2022	4	3	1	1		11	
9 May	2022	18	15	3			12	
10 May	2022	15	11	4			13	
11 May	2022	13	12	1	2		14	
12 May	2022	2	1	1	1		15	
13 May	2022	2	2		2		16	
14 May	2022	1	1		1		17	
15 May	2022	3	3		1		18	
16 May	2022	5	3	2	1		19	
17 May	2022	9	7	2	2		20	
18 May	2022	12	5	7	5		21	
19 May	2022	4	1	3			22	
20 May	2022	3	1	2			23	
21 May	2022					10	24	
22 May	2022					5	25	
23 May	2022					9	26	
24 May	2022	2	1	1			27	
25 May	2022					6	28	
26 May	2022	3		3			29	
27 May	2022	4	2	2	1		30	
28 May	2022	2	2		2		31	
29 May	2022	7	4	3	4		32	
30 May	2022	6	1	5	1		33	
31 May	2022						34	
1 June	2022	2	1	1	1		35	
2 June	2022						36	
3 June	2022	5	4	1	4		37	
4 June	2022	5	2	3	2		38	
5 June	2022	23	6	17	6		39	
6 June	2022	20	4	16	4		40	
7 June	2022	24	4	20	3		41	
8 June	2022	10	3	7	3		42	
9 June	2022	5	2	3	2		43	
10 June	2022					0		No BMB observed
11 June	2022					0		No BMB observed
12 June	2022					0		No BMB observed
Total	2022	324	168	156	49			4 males discarded on 28 April included in totals
15 April	2023					0		No BMB observed
22 April	2023					0		No BMB observed
23 April	2023					2	1	
24 April	2023					0	2	No BMB observed
25 April	2023					1	3	
26 April	2023					1	4	
27 April	2023					5	5	
28 April	2023					30	6	
29 April	2023					4	7	
30 April	2023					4	8	
1 May	2023	2	2			3	9	
2 May	2023					2	10	
3 May	2023	1	1			10	11	
4 May	2023	4	1	3		8	12	
5 May	2023	8	7	1		10	13	
6 May	2023	1	1			6	14	
7 May	2023					2	15	
8 May	2023					16	16	
9 May	2023	3	3			14	17	
10 May	2023	2	1	1		6	18	
11 May	2023	6	6		5	60	19	BMB began going back downstream
12 May	2023	2	1	1		35	20	
13 May	2023					10	21	
14 May	2023	1	1		1	8	22	
15 May	2023					20	23	ARL estimated > 80% males; no anglers present
16 May	2023					70	24	ARL estimated > 80% males; no anglers present
17 May	2023					100	25	ARL estimated > 80% males; no anglers present
18 May	2023					50	26	ARL estimated > 80% males; no anglers present
19 May	2023					0		No BMB observed
20 May	2023					0		No BMB observed
21 May	2023					0		No BMB observed
Total	2023	30	24	6	6			

*#F* number of females, *#M* number of males, *#PSF* number of post-spawn females, *BMB/h* estimate of number of BMB observed per hour at the lakeside water control structure (on days that this was estimated), *MD* migration day number.

In 2022, we again focused monitoring on bigmouth buffalo captured by any means at the lakeside water control structure by recording the date that any bigmouth buffalo was captured, but during this year we used gonadal condition as a proxy to supplement our understanding of movement direction because these two variables were highly associated for data collected in 2021. In 2022 starting on 20 April, we began monitoring the lakeside water control structure for any signs of migrating bigmouth buffalo for at least 1 h each day. No bigmouth buffalo were observed until 28 April 2022 (Table 1), during which monitoring increased to 4 h. On this date bowfishers shot 4 bigmouth buffalo, all of which were males, but these were discarded by bowfishers. On 29 April across 4 h of observations, bigmouth buffalo were observed in aggregations (estimated to be 90% composed of males) on the downstream side of the water control structure prior to swimming through the gates (although none was observed swimming through the gates, just swimming up to the gates and then falling back). Bowfishers and spearfishers did not exploit fish on 29 April 2022 because they were aware the shoreline season had not yet opened. On 30 April 2022 the shoreline bowfishing and spearing season opened and beginning on this date, the spawning migration was monitored for approximately 10–12 h per day for 44 consecutive days (30 April–12 June) until no bigmouth buffalo were observed post-spawn for three consecutive days (10–12 June) and the spawning migration was deemed over (Table 1).

In 2023 we focused monitoring on counts of bigmouth buffalo aggregated immediately downstream or upstream of the lakeside control structure. We conducted counts by tallying the number of bigmouth buffalo observed per hour on each date (Table 1). We also studied any bigmouth buffalo captured by anglers. We monitored the location starting on 15 April and then again on 22 April for at least 1 h. During this timeframe temperatures were still around freezing, and there was extremely high water and no bigmouth buffalo was observed. Water levels were higher in the Mississippi River on 15 April, and because of this, the current had switched direction and was rapidly flowing through the water control structure as it flowed into Rice Lake. However, by 22 April the current was at approximately equilibrium and by 23 April water was flowing out of Rice Lake again as it normally does. Rice Lake predominantly serves as an outlet of water, but it is not uncommon for the current to switch during times of high water and uneven rain^29,32^. Then, for the next 29 consecutive days (23 April–21 May) the lakeside water control structure was monitored for 1–8 h per day. Again, the monitoring effort concluded when no bigmouth buffalo was observed for three consecutive days post-spawn and the spawning migration was determined over (Table 1).

### Fish collection from recreational exploitation

During the monitoring period we obtained bigmouth buffalo taken from the lakeside water control structure by recreational bowfishers, spearfishers and hook & line anglers. Anglers were fishing on their own recreational accord, and fish carcasses were retrieved after an angler captured a fish. All captured bigmouth buffalo were retrieved by our research team, except for 4 males shot on 28 April 2022 (see paragraph above). We obtained fish typically within 10 min of collection by the angler.

### Body dissections

**Figure 2:**
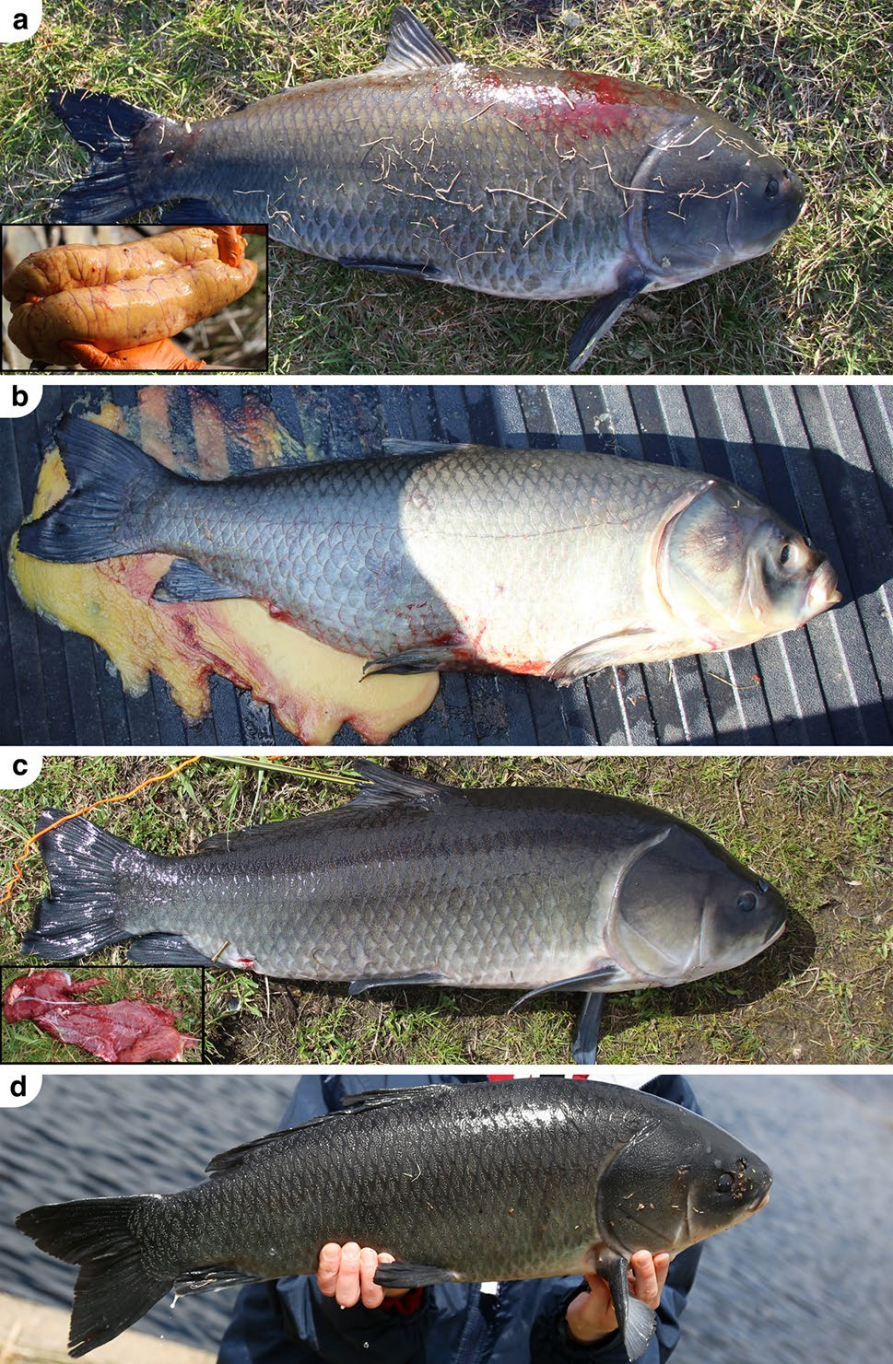
Spawning phenotypes of bigmouth buffalo from Rice Lake National Wildlife Refuge, Minnesota. (**a**) A 90-year-old gravid female (80.1 cm TL; 8.41 kg in mass; GSI of 0.174) captured on 4 May 2021 while moving upstream towards Rice Lake (with a convex ventral margin and, in the inset, pre-spawn ovaries). (**b**) A 79-year-old gravid female (91.3 cm; 9.55 kg) captured on 3 May 2021 while moving upstream to Rice Lake (which began releasing eggs at capture). (**c**) A post-spawn 64-year-old female (81.6 cm; 7.9 kg; GSI of 0.027) captured on 4 May 2021 while moving downstream from Rice Lake; (with a concave ventral margin and, in the inset, spawned out ovaries). (**d**) A tuberculate 84-year-old male (68.6 cm; 4.76 kg) captured on 7 May 2021 while moving downstream from Rice Lake.

For each bigmouth buffalo retrieved from anglers we photographed both sides of the fish, noted pigmentation characteristics, measured fish size, dissected the individual’s gonadal tissue to determine sex and spawning state (Fig. 2), and extracted the otoliths. All animals were treated in accordance with the animal protocols A17007 and 2007-38272A approved by the University of Minnesota Duluth Institutional Animal Care and Use Committee, and all procedures were carried out in accordance with all relevant guidelines and regulations. This study complies with the ARRIVE (Animal Research: Reporting of In Vivo Experiments) guidelines^34^. After obtaining photographs of the fish, we noted the presence/absence of orange and black pigmentation on scales or skin, and presence/absence of white markings along the fins. We quantified size by wet mass (± 0.01 kg) and total length (± 1 mm) either immediately after the fish was landed, or within a few hours after the fish was captured (and these specimens were immersed in water prior to obtaining mass). In 2021, we measured gonad mass (± 0.01 kg) for all pre-spawn females, a subset of post-spawn females, and a subset of males. In 2022–2023, we measured gonad mass (± 0.01 kg) for all individuals regardless of sex or spawning state. For all individuals for which gonad mass was measured, we calculated the gonadosomatic index (GSI = gonad mass/ total body mass). Following dissection, we categorized females as pre-spawn if the ovaries were fully gravid with eggs (Fig. 2a) or post-spawn if the ovarian membranes were collapsed, were reddish in color (as opposed to yellow-orange) and contained only residual eggs because the individual had spawned out (Fig. 2c). We categorized males as pre-spawn (GSI > 0.040) or post-spawn (GSI ≤ 0.040) based on the distribution of male GSI across the spawning season. After extraction we placed otoliths immediately in microvials pre-filled with distilled water.

### Seining for young-of-the-year post spawn

During years 2022 and 2023 we sampled young-of-the-year bigmouth buffalo near the lakeside water control structure at regular intervals from late June to early November. We sampled for young-of-the-year along several ~ 25 m long transects (passes) of various aquatic habitat types (shoreline, river channel, pools adjacent to gates) where spawning bigmouth buffalo have been observed. We seined using a 15.24 m × 1 m bag seine with 0.635 cm mesh. We seined approximately weekly from late June (28 June 2022, 30 June 2023), the earliest approximate dates for bigmouth buffalo fry to become mobile based on observed water temperatures^4^ until early November (2 November 2022, 11 November 2023) when Rice Lake began to ice-over. In 2022, we sampled six locations on each date (a single site immediately upstream of the control structure, and five sites immediately downstream of the control structure) with a single pass of the seine during each transect for a total of six seine hauls on each sampling date. In 2023, we sampled the same upstream site as 2022, but included two more immediately-upstream sites because the gates of the control structure were closed (fish passage downstream was not possible). Again, we made a single pass of the seine during each transect. Starting on 15 July 2023, we only sampled the upstream sites (due to the gates being closed) and varied the number of seine hauls from 3 to 4 transects per session. After the gates were re-opened on 2 November 2023, nine transects were taken on the last sampling date (four upstream sites and the five downstream sites). After each seine pull, captured fish were photographed, identified to species and counted, and young-of-the-year bigmouth buffalo were measured for total length (± 1 mm), body depth (± 0.01 mm), and wet mass (± 0.01 g). We used the count totals and number of seine passes to estimate catch per unit effort (CPUE, fish per pass) of young-of-the-year bigmouth buffalo, and other species, over time.

### Otolith analysis

In the lab we processed otoliths to obtain photographs of their whole structure and thin sectioned them to determine age. For methodological protocol see^4–7^. If an additional otolith thin section was necessary for a given individual to resolve uncertainty in age quantification, then another otolith from that individual was sectioned until it was resolved.

### Quantification of water level

We used water level data from late-April to late-June, and lakeside control structure status from late April to early November in Rice Lake from 2021 to 2023, along with historical water level records for Rice Lake to quantify habitat conditions. Water level data (meters above sea level on the downstream side of the water control structure) was logged by RLNWR headquarters every 2–5 days during the timeframe of 28 April–13 June 2021. We also recorded the status (open, indicating water can flow between the outlet and Rice Lake; closed, indicating Rice Lake is closed to the outlet) of the lakeside control structure during the monitoring period. In both 2021 and 2022 the lakeside structure remained open year-round. However in 2023, the lakeside control structure was open until 12 June 2023, at which point it was closed because of an expected drought. The gates remained closed until 2 November 2023 and were opened thereafter. We also obtained April through June water level data from RLNWR headquarters (Walt Ford, RLNWR manager, data available upon request January 2024) for all years this was additionally available (23 years during 1990–2018).

### Statistical analysis

For analysis of date of capture, we used a two-way analysis of variance (ANOVA) to analyze the relationship between sex, movement direction, and the interaction between sex and movement direction for bigmouth buffalo collected in 2021. We also used two-way ANOVA to analyze the relationship between sex, spawning classification, and the interactions between sex and spawning classification on the day of capture for bigmouth buffalo collected in 2022.

For analysis of GSI data, we used a two-sample t-test to analyze whether mean GSI significantly differed between females and males, by spawning classification (pre- or post-spawn). We also used analysis of covariance (ANCOVA) to assess the effects of day, sex, and the interaction between day and sex on GSI. We used ANCOVA to evaluate allometry of gonad mass versus body mass for bigmouth buffalo more than 50 years old. We used multiple logistic regression analysis to quantify the duration and central timing of the spawning season in each of years 2021–2023 (via female spawning state), and ANCOVA to quantify rate of water level decline each spring.

We used ANCOVA to test if age of bigmouth buffalo was related to day of capture, collection year, or the interaction between day of capture and collection year. We calculated the average coefficient of variation (CV) across age reader scores to quantify age estimate precision^35^. We analyzed recruitment success using contingency analysis^4,5,7^. Using sample data, we defined “evidence of recruitment” categorically (Yes/No) for each year 1926–2018, based on whether a 2021–2023 collected bigmouth buffalo was estimated to be from that respective year class. We used 1926 as the starting point because that is the earliest bigmouth buffalo year class in the sample. We used 2018 as the end point because bigmouth buffalo males are known to reach the onset of sexual maturity (at the population level) by an age of 5–6 years in Minnesota near this latitude^6^. Therefore by 2023, bigmouth buffalo from the 2018 year class (5 years old) would likely be migrating if fish from that year class successfully recruited to the population. We then tested whether evidence of recruitment in a given year is independent of evidence of recruitment the previous year to analyze recruitment episodicity. We also used contingency analysis to evaluate evidence of recruitment success (Yes/No) in the pre (1926–1952) versus post water control structure era (1953–2018). We also ran this analysis using a pre-era spanning 1895–1952 because bigmouth buffalo can live more than 125 years^4^.

We used ANCOVA to analyze growth in TL of asymptotically mature (> 50 years old) bigmouth buffalo with effects for sex, age, and the interaction of sex and age. For analysis of spot pigmentation, we used contingency analysis to determine if orange spot presence was associated with collection year. We also conducted a chi-square test for association between the presence/absence of white-edged fins and orange spot presence/absence, for individuals greater than 50 years old.

For bigmouth buffalo young-of-the-year data we used ANCOVA to analyze CPUE across day of year with additional variables including year and the interaction between day of year and year. We used linear regression to analyze trends in bigmouth buffalo young-of-the-year growth in body depth in comparison to the maximum prey size of young-of-the-year northern pike (in body depth)^36^ collected at RLNWR. We used JMP® Pro Version 17 (SAS Institute, Inc., Cary, NC 1989–2024) software for statistical analyses.

## Results

**Figure 3:**
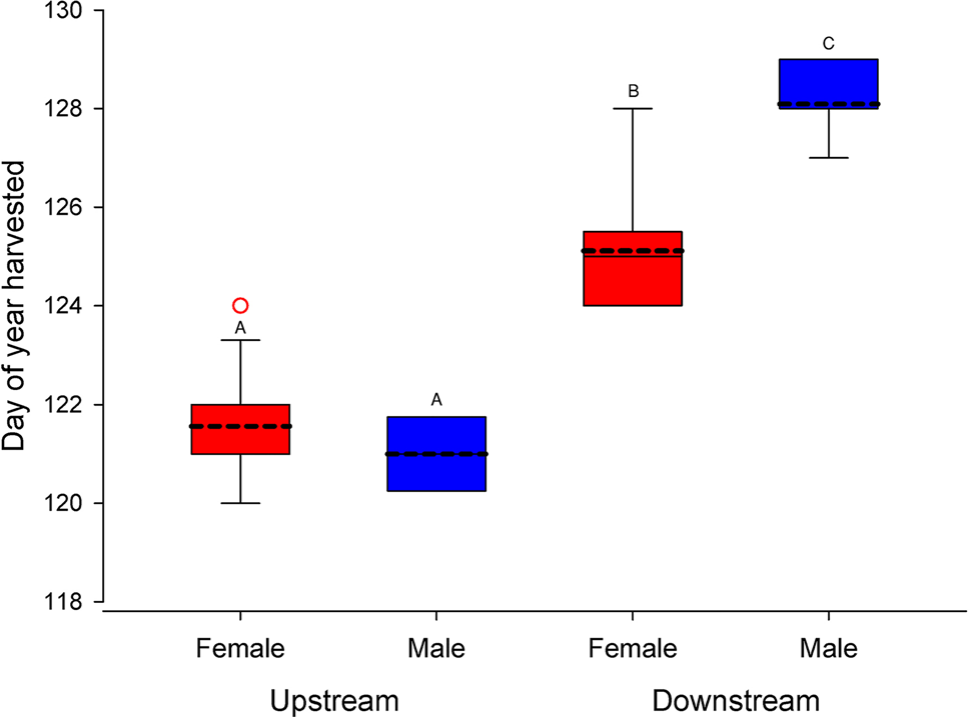
Distribution of day of capture for bigmouth buffalo captured by anglers in 2021 with respect to movement direction (upstream versus downstream) and sex (female versus male). Boxes illustrate 1st quartile, median, and 3rd quartile; whiskers show minimum and maximum dates; dashed line indicates least-square mean and open circle indicates outlier. Different upper case letters indicate significant differences in least-square means as determined by a Tukey test following two-way ANOVA.

In 2021 bigmouth buffalo moving upstream were captured significantly earlier in the year compared to fish moving downstream. We observed the direction of movement when initially shot, speared or hooked by anglers for 40 bigmouth buffalo in 2021, and 90% of the variation in day of capture (F_3,36_ = 103.63, *P* < 0.0001, *R*^2^ = 0.90) was explained by a model with sex (F_1,36_ = 10.83, *P* = 0.0022, *R*^2^ = 0.03), movement direction (F_1,36_ = 209.89, *P* < 0.0001, *R*^2^ = 0.61) and the interaction of sex and movement direction (F_1,36_ = 23.27, *P* < 0.0001, *R*^2^ = 0.07). A Tukey test indicated the least square means for day (of the year) of capture for upstream migrating females (121.56 ± 0.26) and males (121.00 ± 0.52) were not significantly different, but were significantly earlier than the mean for downstream migrating females (125.11 ± 0.34), which was significantly earlier than the mean for downstream migrating males (128.09 ± 0.31) (Fig. 3).

**Figure 4:**
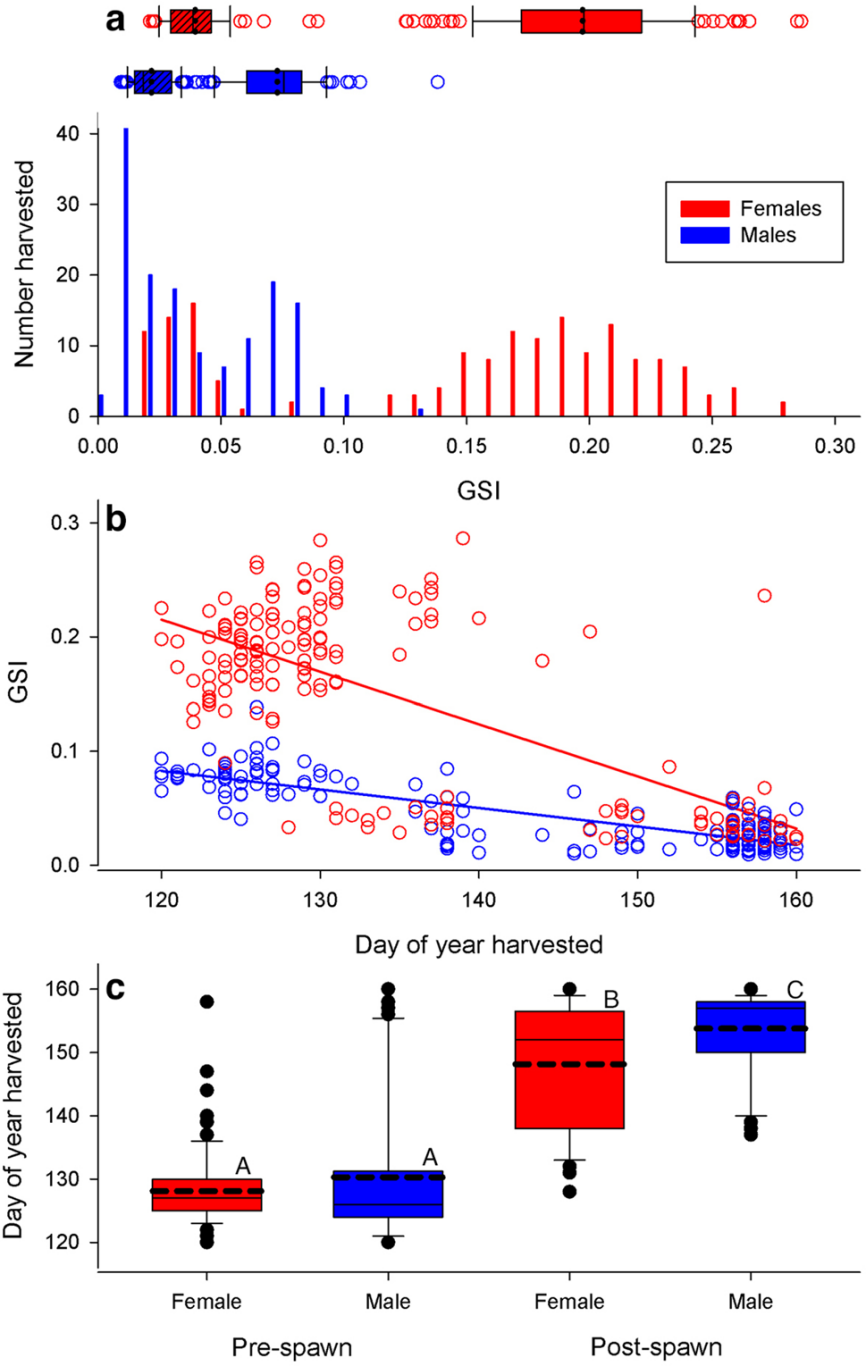
(**a**) Distribution of gonadosomatic index (GSI) for female (red) and male (blue) bigmouth buffalo captured by anglers in 2022. The bimodal distribution for each sex is summarized with box & whisker plots indicting minimum, 1st quartile, median, 3rd quartile and maximum values, with dashed line indicating least-square mean for each mode. Hatched box plots represent pre-spawn individuals, solid box plots represent post-spawn individuals). (**b**) GSI declined significantly with day harvested, sex, and their interaction. (**c**) When spawning status (pre-spawn versus post-spawn) was assigned by GSI, least-square mean day of harvest differed significantly for females and males post-spawn, but not pre-spawn (as determined by a Tukey test following two-way ANOVA, as indicated by upper case letters).

For bigmouth buffalo captured in 2022, GSI was bimodal, declined with day of the year and indicated pre-spawn males and females were captured earlier than post-spawn females, which were captured earlier than post-spawn males. GSI was bimodal for both females (range 0.089–0.286 for pre-spawn females, and 0.021–0.086 for post-spawn females, lower mode < 0.087, *n* = 168) and males (range 0.009–0.138, lower mode < 0.040, *n* = 152). Pre-spawn female GSI was significantly greater (mean ± SE 0.197 ± 0.003) than for pre-spawn males (0.073 ± 0.002) (F_1,187_ = 702.48, *P* < 0.0001, *R*^2^ = 0.79), and post-spawn female GSI was significantly greater (mean ± SE 0.039 ± 0.002) than for post-spawn males (0.022 ± 0.001) (F_1,129_ = 92.75, *P* < 0.0001, *R*^2^ = 0.42) (Fig. 4a). GSI declined significantly (F_3,316_ = 251.46, *P* < 0.0001, *R*^2^ = 0.70) with day (F_1,316_ = 263.99, *P* < 0.0001, *R*^2^ = 0.25), sex (F_1,316_ = 229.49, *P* < 0.0001, *R*^2^ = 0.21) and day*sex interaction (F_1,316_ = 59.90, *P* < 0.0001, *R*^2^ = 0.06) across the monitoring period, but the rate of decline was significantly greater for females (slope of − 0.0046 ± 0.0002) compared to males (− 0.0031 ± 0.0002) (Fig. 4b). Assuming post-spawn individuals have a GSI below the threshold of 0.087 for females, and 0.040 for males, over 67% of the variation in day of capture (F_2,317_ = 325.88, *P* < 0.0001, *R*^2^ = 0.67) was explained by sex (F_1,316_ = 15.44, *P* = 0.0001, *R*^2^ = 0.02), and pre/post-spawn (F_1,316_ = 548.72, *P* < 0.0001, *R*^2^ = 0.57), and a Tukey test indicated the least square means for day of capture for pre-spawn females (128.14 ± 0.74) and pre-spawn males (130.30 ± 0.96) were not significantly different, but were significantly earlier than the mean day of capture for post-spawn females (147.66 ± 1.13), which was significantly earlier than the mean day of capture for post-spawn males (153.78 ± 0.88) (Fig. 4c).

Gonad mass versus body mass of asymptotically mature (> 50 years old) bigmouth buffalo revealed positive allometry. ANCOVA indicated that spawning classification (pre- vs. post-spawn), log-transformed mass, the interaction of spawning classification and log-transformed mass, collection year, and age explained more than 94% of the variation in log-transformed gonad mass for females (*F*_6,200_ = 538.03, *P* < 0.0001, *R*^2^ = 0.94). Log-transformed mass (*F*_1,200_ = 303.93, *P* < 0.0001, *R*^2^ = 0.09) and spawning classification (*F*_1,200_ = 2112.85, *P* < 0.0001, *R*^2^ = 0.62) were significant effects, but age (*P* = 0.4603), collection year (*P* = 0.2923), and the interaction of spawning classification and log-transformed body mass (*P* = 0.5666) were not. We analyzed a post hoc model with only log-transformed mass (*F*_1,206_ = 392.72, *P* < 0.0001, *R*^2^ = 0.11) and spawning classification (*F*_1,206_ = 2317.22, *P* < 0.0001, *R*^2^ = 0.67) and more than 94% of the variation was explained (*F*_2,204_ = 1616.85, *P* < 0.0001, *R*^2^ = 0.94). The slope coefficient ± SE for the post hoc model = 1.49 (± 0.08) indicated that gonad mass increases proportional to (body mass)^1.49^ as asymptotically mature female bigmouth buffalo increase in size. Likewise for males, ANCOVA indicated that spawning classification (pre- vs. post-spawn), log-transformed mass, the interaction of spawning classification and log-transformed mass, collection year, and age explained more than 80% of the variation in log-transformed gonad mass for males (*F*_6,161_ = 111.34, *P* < 0.0001, *R*^2^ = 0.81). Again, log-transformed mass and spawning classification were significant effects, but age (*P* = 0.2531), collection year (*P* = 0.9623), and the interaction of spawning classification and log-transformed body mass (*P* = 0.1620) were not. We analyzed a post hoc model with only log-transformed mass (*F*_1,165_ = 54.66, *P* < 0.0001, *R*^2^ = 0.07) and spawning classification (*F*_1,165_ = 508.02, *P* < 0.0001, *R*^2^ = 0.61) and more than 80% of the variation was explained (*F*_2,165_ = 332.35, *P* < 0.0001, *R*^2^ = 0.80). The slope coefficient ± SE for the post hoc model = 1.19 (± 0.16) indicated that gonad mass increases proportional to (body mass)^1.19^ as asymptotically mature male bigmouth buffalo increase in size.

**Figure 5:**
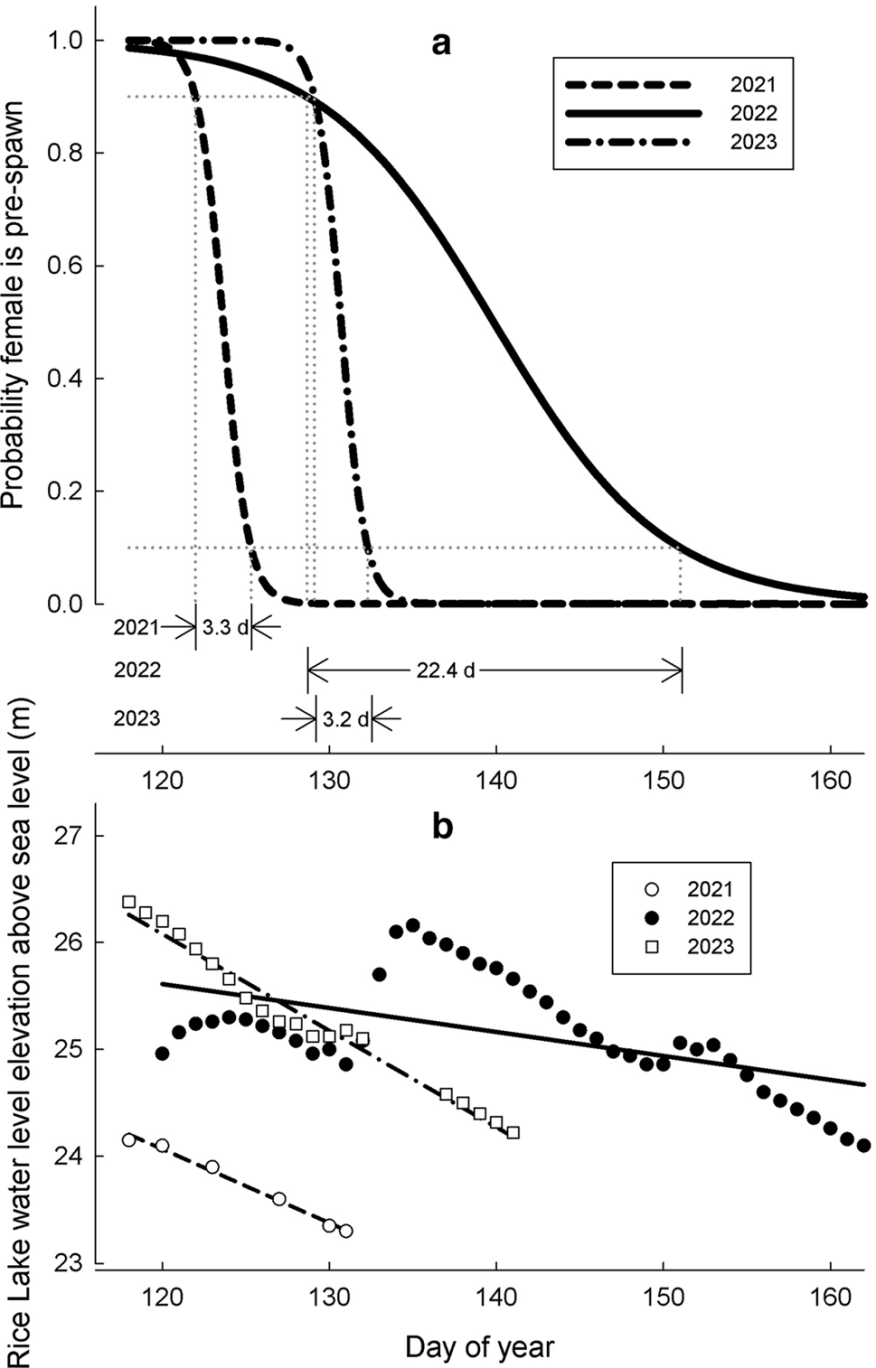
Bigmouth buffalo *Ictiobus cyprinellus* spawning phenology at Rice Lake National Wildlife Refuge across years. Interannual differences in (**a**) timing and duration of the spawning season for female bigmouth buffalo at Rice Lake and (**b**) rate of water level recession in Rice Lake. Probability of female reproductive state (pre- vs post-spawn) was modeled by logistic regression with effects of year, day captured and the interaction between year and day captured. Start of the spawning season was defined as the day for which probability of pre-spawning state at capture was 0.9 and end of the season was defined as the day for which probability of pre-spawning state at capture was 0.1, with duration defined as the difference between the start and end days.

The duration and central timing of bigmouth buffalo captures varied significantly across years, and so did peak water level and the rate of water level decline. The total spawning migration phenology of bigmouth buffalo at Rice Lake National Wildlife Refuge spanned 15 days in 2021, 43 days in 2022, and 26 days in 2023 (Table 1, Fig. 5). Multiple logistic regression analysis of pre/post-spawn classification of captured bigmouth buffalo as a function of day of year, year, sex, and the interactions of year*day, sex*year, and sex*day revealed significant effects in all but the sex*day term (*P* = 0.1938). In a post-hoc model excluding the sex*day term (*χ*^2^ = 295.23, *df* = 8, *P* < 0.0001,* R*^2^ = 0.57, *n* = 390), approximately 57% of the variation in pre/post-spawn classification could be explained by day of year (*χ*^2^ = 48.00, *df* = 1, *R*^2^ = 0.16, *P* < 0.0001), year (*χ*^2^ = 22.93, *df* = 2, *R*^2^ = 0.08, *P* < 0.0001), year*day (*χ*^2^ = 20.2, *df* = 2, *R*^2^ = 0.07, *P* < 0.0001), sex (*χ*^2^ = 14.20, *df* = 1, *R*^2^ = 0.05, *P* = 0.0002), and sex*year (*χ*^2^ = 11.24, *df* = 2, *R*^2^ = 0.04, *P* = 0.0038) (Fig. 5a). Using this logistic model and assuming females control the pace of the spawn, we predicted the spawning mid-point (P = 0.5 for post-spawn female) to be day 123.7 (3 May) 95% CI [122.5–125.1] in 2021, 139.8 (19 May) [137.2–142.9] in 2022, and 130.7 (10 May) [129.7–131.7] in 2023. Using P = 0.1 and 0.9 to compare the middle 80% of the spawning phenology for females, we found that this lasted approximately 3.3 days in 2021 (days 122.0–125.3), 22.4 days in 2022 (days 128.7–151.0), and 3.2 days in 2023 (days 129.1–132.3) (Fig. 5a). Additionally, ANCOVA indicated that 85% of the variation in water level (*F*_5,68_ = 75.56, *P* < 0.0001, *R*^2^ = 0.85) was explained by day of year (*F*_1,72_ = 39.86, *P* < 0.0001, *R*^2^ = 0.09), year (*F*_2,71_ = 30.67, *P* < 0.0001, *R*^2^ = 0.14), and the interaction of day*year (*F*_2,71_ = 45.26, *P* < 0.0001, *R*^2^ = 0.20) (Fig. 5b). The peak spring water level observed on 16 April 2021 (before bigmouth buffalo were observed in the system) was at the 30^th^ percentile for spring peaks at this site from 1990 to 2023). The spring peak that occurred in 2022 (which was on 15 May well after bigmouth buffalo were first observed in the system) was at the 73rd percentile, whereas the spring peak in 2023 occurred on 15 April (before bigmouth buffalo were observed in the system) and was a spring peak at the 99th percentile.

**Figure 6:**
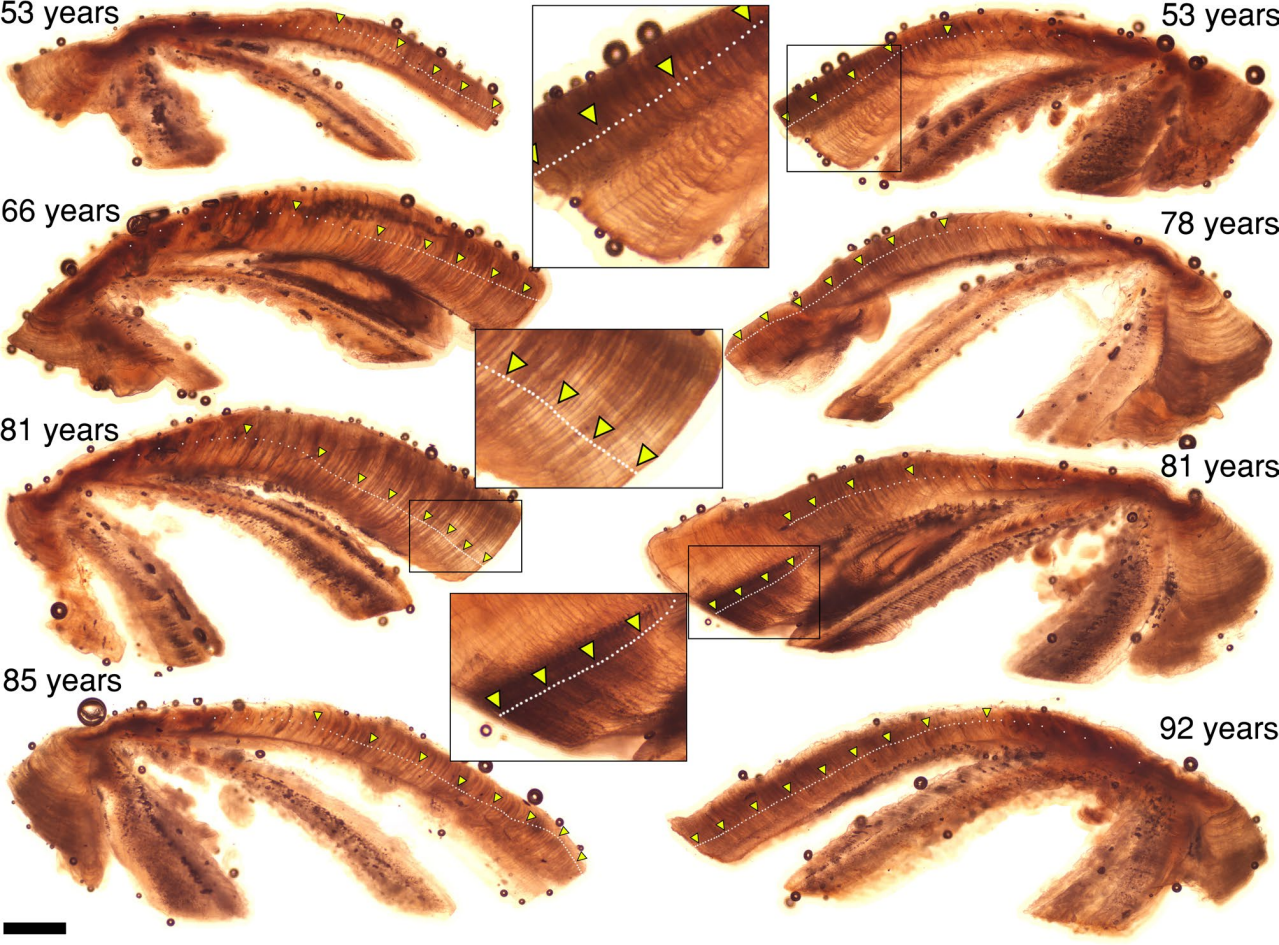
Thin-sectioned asteriscus otoliths from bigmouth buffalo *Ictiobus cyprinellus* captured at Rice Lake National Wildlife Refuge, Minnesota. Examples are of 8 different individual fish ranging from 53 to 92 years old. Insets are of the last several decades of otolith growth for the 53-year-old individual on the upper right, and the 81-year-old individuals. White dots note annuli, triangles mark decades. Scale bar = 600 μm (does not apply to insets).

**Figure 7:**
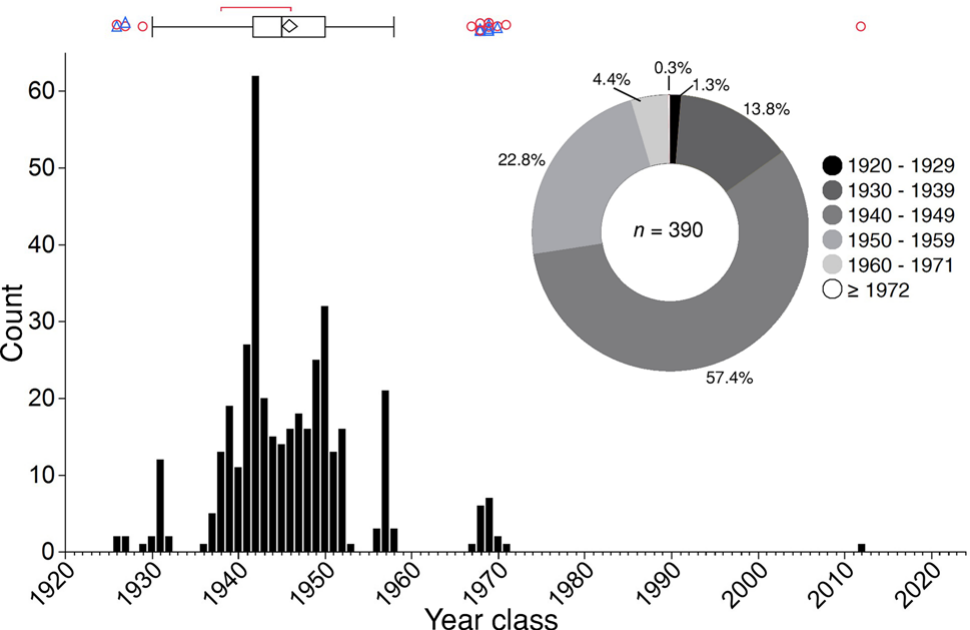
Year class distribution of bigmouth buffalo *Ictiobus cyprinellus* collected during 2021–2023 from Rice Lake National Wildlife Refuge, Minnesota. Year classes range from 1926 to 2012, with 99.74% of individuals hatched before 1972. The 1942 year class is most abundant, and three other year classes exhibit distinct modes (1931, 1950, and 1957). The pie chart shows the distribution of year classes across decades: 1920s, 1930s, 1940s, 1950s; and two recent eras: 1960–1971, and post 1971. A box-and-whisker plot summarizes the data, with interquartile range, median, mean diamond (width of diamond is 95% CI of mean), shortest half of data (red bracket), and statistical outliers (females = red open circles, males blue open triangles).

We estimated ages and calculated year classes of all 390 angler-retrieved bigmouth buffalo in this study. We found that 99.7% of individuals hatched prior to 1972, that is, greater than 52 years old as of 2024 (e.g., Fig. 6). For this population of bigmouth buffalo as of 2024, the modal age is 82, the median is 79, the mean is 78, and the interquartile range is from 74 to 82 years (1942–1950 year classes; Fig. 7). Approximately 83.1% of the bigmouth buffalo in this population are estimated to have hatched during the 18-year span of 1936–1953 (Fig. 7). ANCOVA indicated that age of fish was not explained (*F*_5,384_ = 1.18, *P* = 0.3160, *R*^2^ = 0.02) by year (*P* = 0.5872), day of capture (*P* = 0.6110), or the interaction of year and day of capture (*P* = 0.5004). The overall between-reader aging precision had a coefficient of variation (CV) mean of 3.9% and a median of 3.6%. Size of these bigmouth buffalo ranged from 66.5 to 104.0 cm TL and 3.90–18.20 kg in mass, and there were 217 females and 173 males. The smallest fish was not the youngest, and the largest fish was not the oldest (Table 2). Across all specimens (*n* = 390), an aggregate total of 393 otolith thin sections was produced.

**Table 2:** Demographic characteristics of the smallest, largest, youngest, oldest, least, and most fecund bigmouth buffalo *Ictiobus cyprinellus* collected from Rice Lake National Wildlife Refuge in 2021–2023 (*n* = 217 females, 173 males). Size (cm) in total length (TL), mass in kg, and gonad (G) in g; GSI = gonadosomatic index expressed as a relative frequency.

Species	Category	Sex	TL	Mass	Age	G	GSI	Note	Capture Date
*I. cyprinellus*	Smallest TL	M	66.5	4.18	71				1 May 2021
*I. cyprinellus*	Largest TL	M	87.6	8.72	73	400	0.046		8 May 2021
*I. cyprinellus*	Smallest mass	M	69.8	3.90	73	59	0.015		5 June 2022
*I. cyprinellus*	Largest mass	M	85.6	9.36	81	86	0.009		6 June 2022
*I. cyprinellus*	Youngest	M	76.5	6.58	53	508	0.077		1 May 2022
*I. cyprinellus*	Youngest	M	70.7	4.98	53	417	0.084		7 May 2022
*I. cyprinellus*	Youngest	M	73.0	5.35	53	109	0.009		9 June 2022
*I. cyprinellus*	Youngest	M	78.6	7.42	53	376	0.051		10 May 2023
*I. cyprinellus*	Oldest	M	77.6	6.81	96	635	0.093		30 April 2022
*I. cyprinellus*	Smallest TL, mass, & youngest	F	67.8	4.76	10	168	0.035	Spawned out	17 May 2022
*I. cyprinellus*	Smallest mass, > 50 year old F	F	81.6	6.82	71	159	0.023	Spawned out	28 May 2022
*I. cyprinellus*	Largest TL, & mass	F	104.0	18.20	80	1229	0.068	Spawned out	7 June 2022
*I. cyprinellus*	Smallest gravid F TL	F	78.0	9.21	82	1619	0.176	Gravid	1 May 2023
*I. cyprinellus*	Smallest gravid F mass	F	79.6	7.40	83	1039	0.140	Gravid	3 May 2022
*I. cyprinellus*	Largest gravid F TL & mass	F	101.6	17.46	72	3706	0.212	Gravid	9 May 2022
*I. cyprinellus*	Oldest	F	84.5	8.43	95			Spawned out	5 May 2021
*I. cyprinellus*	Oldest	F	83.2	7.80	95	1148	0.147	Gravid	3 May 2022
*I. cyprinellus*	Highest GSI	M	79.1	6.86	80	948	0.138		6 May 2022
*I. cyprinellus*	Lowest gravid GSI	F	87.5	9.38	72	839	0.089	Gravid	4 May 2022
*I. cyprinellus*	Highest gravid GSI	F	96.9	16.48	91	4717	0.286	Gravid	19 May 2022
	Median of each category	M	76.1	6.07	78	238	0.040		
	Median of each category	F	88.7	9.74	79	381	0.038	Spawned out	
	Median of each category	F	88.5	11.17	77	2180	0.194	Gravid	

Bigmouth buffalo recruitment is episodic at Rice Lake National Wildlife Refuge and declined significantly after construction of the lakeside control structure in 1953. Evidence of bigmouth buffalo recruitment observed in one year was significantly more likely when evidence of recruitment was observed in the previous year (*χ*^2^ = 53.3, df = 1, *n* = 92, *P* < 0.0001, *R*^*2*^ = 0.44). That is, if evidence of recruitment was not observed in a year, then it was 90% likely to not be observed again the following year. Conversely, if evidence of recruitment was observed in a year, then it was 84% likely to be observed the following year. In addition, evidence of recruitment for the period prior to completion of the lakeside control structure (years 1926–1952) was significantly more likely compared to the period after completion of the control structure (years 1953–2018) (*χ*^2^ = 42.18, *df* = 1, *n* = 93, *P* < 0.0001, *R*^*2*^ = 0.35). In a post-hoc comparison we included years 1895–1952 for the period prior to completion of the control structure, and evidence of recruitment for the period prior to completion of the control structure was still significantly more likely compared to the period after completion (*χ*^2^ = 9.64, *df* = 1, *n* = 124, *P* = 0.0019, *R*^*2*^ = 0.07).

Evidence indicates asymptotically mature bigmouth buffalo (> 50 years old) grow at a rate of approximately 0.1 cm per year within both sexes. ANCOVA indicated that sex and age explained more than 65% of the variation in total length (*F*_3,385_ = 245.38, *P* < 0.0001, *R*^2^ = 0.66), but that the interaction between sex and age was not significant (*P* = 0.7858). We analyzed a post hoc model with only sex (*F*_1,386_ = 730.79, *P* < 0.0001, *R*^2^ = 0.65) and age (*F*_1,386_ = 13.01, *P* = 0.0004, *R*^2^ = 0.01) and more than 65% of the variation was explained (*F*_2,386_ = 368.91, *P* < 0.0001, *R*^2^ = 0.66). The slope coefficient ± SE for the post hoc model = 0.11 (± 0.03) indicated that both male and female bigmouth buffalo > 50 years old increase in TL at a rate of 0.1 cm per year, and that females are on average 6.5 (± 0.2) cm longer than males. Overall, asymptotically mature bigmouth buffalo females varied by 26.0 cm in TL (78.0–104.0 cm, sd = 5.1), and males by 21.1 cm TL (66.5–87.6 cm, sd = 4.4). The smallest and largest male varied by 5.46 kg (3.90–9.36 kg), smallest and largest pre-spawn female by 10.06 kg (7.40–17.46 kg), and the smallest and largest post-spawn female (not including the single individual younger than 51 years) varied by 11.38 kg (6.82–18.20 kg), yet these individuals were close in age (71–83 years) and did not represent the youngest or oldest individuals (Table 2).

**Figure 8:**
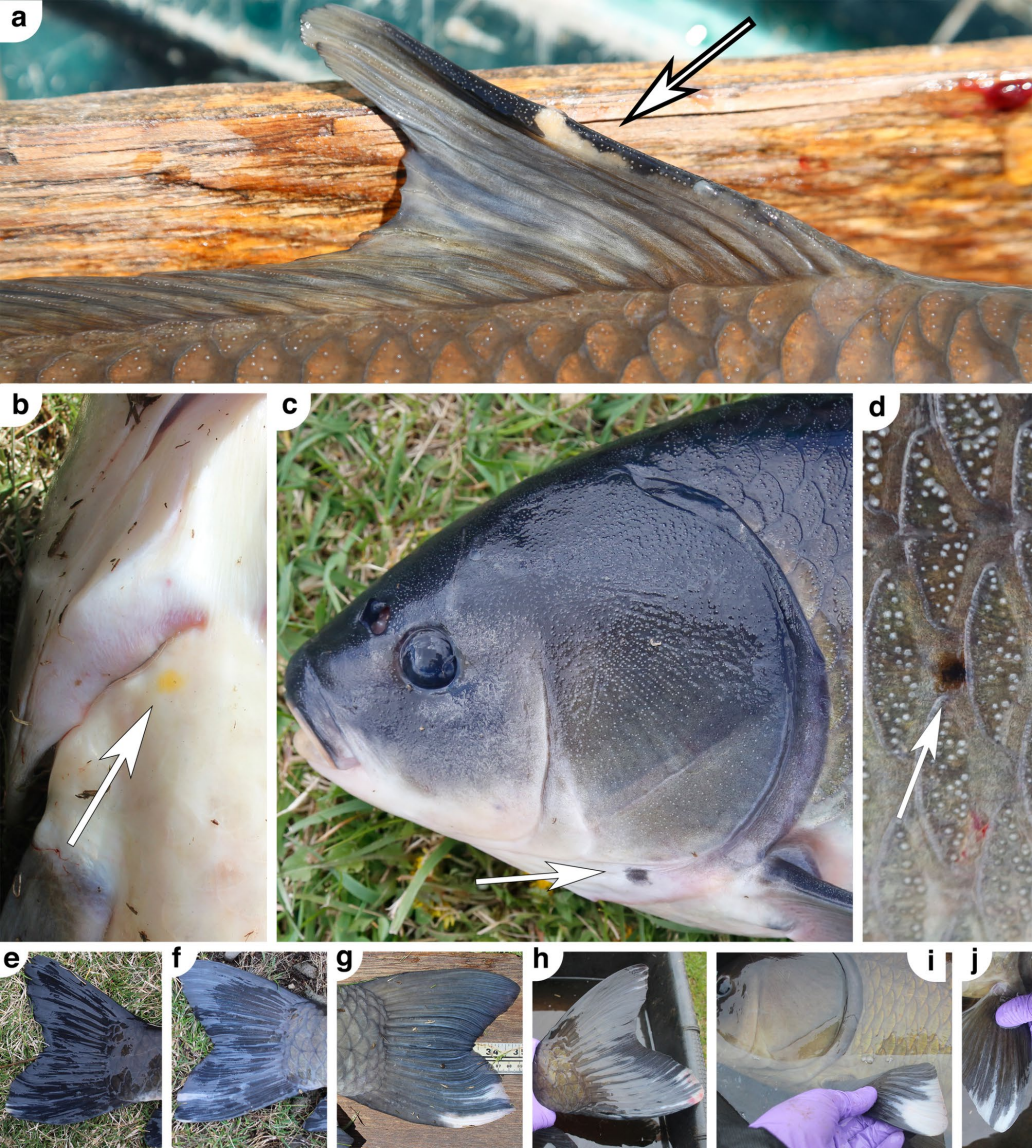
Example black and orange spotting, as well as white-edged fins on bigmouth buffalo *Ictiobus cyprinellus* from Rice Lake National Wildlife Refuge. (**a**) Black pigmentation on the leading dorsal fin ray of this 71-year-old male, which also had a translucent light-orange area. (**b**) An orange spot was located on the ventral anterior of this 64-year-old female. (**c**) A black spot on this 84-year-old male, as well as (**d**) a nested black-orange spot on this fish’s right side (note the rust-like orange peripheral region of the spot). (**e**–**h**) Four individuals (93, 72, 81, 79 years old, respectively) with various degrees of white-pigmentation in the ventral region of the caudal fin. (**i**, **j**) More views of the 79-year-old female first shown in h, which also had pronounced white pigmentation (or depigmentation) in its pectoral fins.

Bigmouth buffalo from Rice Lake National Wildlife Refuge exhibit phenotypic variation indicative of an old-growth population. More than 99% of bigmouth buffalo (389 of 390) had evidence of black spots and 58% (226 of 390) had evidence of orange spots (e.g., Fig. 8a–d). There was a single individual that had no such markings (black or orange), and this was the 10-year-old spawned-out female collected in 2022 (Table 2). The proportion of individuals with orange spots significantly varied across years (*χ*^2^ = 22.02, *df* = 2, *n* = 389, *P* < 0.0001, *R*^2^ = 0.04). Specifically, the observed proportion in 2021 = 0.90, in 2022 = 0.54, and in 2023 = 0.57.

In east-central Minnesota a white-edged fin phenotype of bigmouth buffalo exists. Across all 390 adult bigmouth buffalo, 13 individuals (3.3%) exhibited white-edged fins (e.g., Fig. 8e–j). These consisted of 9 females that ranged from 72 to 93 years old, and 4 males that ranged from 66 to 80 years old. The most common area for this white pigmentation was on the ventral region of the caudal fin (11 of 13 individuals; Fig. 8e–h). The second most common area was on the pectoral fins (6 of 13 individuals; Fig. 8i,j). Three individuals had white pigmentation in the dorsal region of their caudal fin (Fig. 8h), and two individuals had white-edged pelvic fins. When present on pectoral fins (*n* = 6 individuals) or pelvic fins (*n* = 2 individuals), it always occurred on both the left and right fin of each type to various extents (Fig. 8i,j). On four individuals, white pigmentation was noted on multiple fin types (Fig. 8h–j). Of the 13 individuals, the anal fin and the dorsal fin were always normal, never exhibiting white pigmentation. The presence of white markings on bigmouth buffalo was not related to orange spot pigmentation (*χ*^2^ = 0.78, *df* = 1, *n* = 389, *P* = 0.3785, *R*^2^ = 0.00).

**Figure 9:**
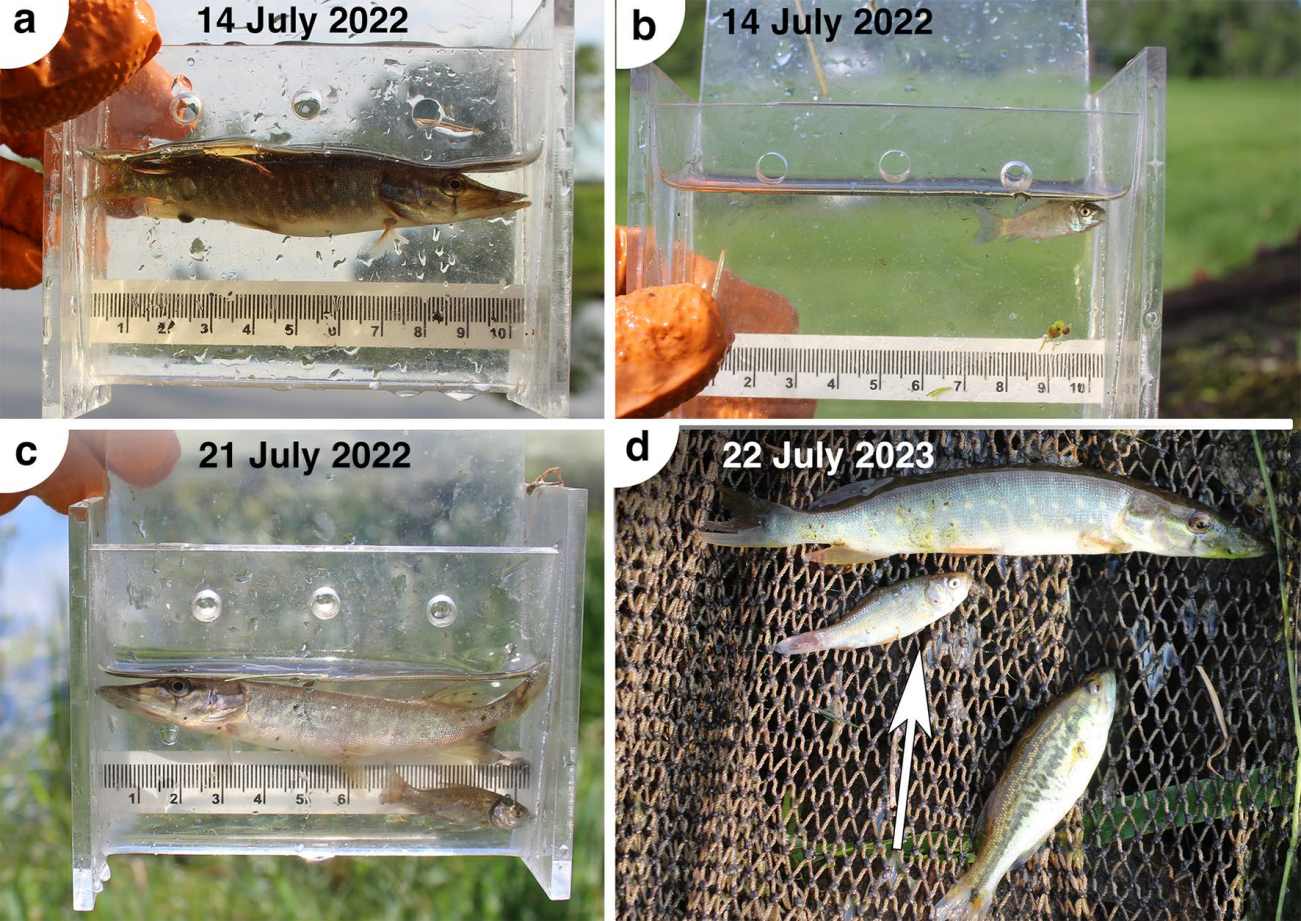
Examples of young-of-the-year northern pike *Esox lucius* and bigmouth buffalo *Ictiobus cyprinellus* collected from Rice Lake National Wildlife Refuge in 2022 and 2023. (**a**, **b**) A young-of-the-year northern pike and bigmouth buffalo collected on 14 July 2022. (**c**) A young-of-the-year northern pike and bigmouth buffalo collected on 21 July 2022. (**d**) A young-of-the-year northern pike, bigmouth buffalo (arrow), and largemouth bass *Micropterus salmoides* collected on 22 July 2023. Bigmouth buffalo young-of-the-year were never captured in regular beach seine sampling after 28 July in 2022, and only once post-July (on 25 August) in 2023, however, northern pike young-of-the-year were captured until late in the sampling season both years: 28 October in 2022 and 2 November in 2023 (Tables 3 & 4). Scale bar integers indicate 1 cm increments, images shown at same scale.

**Figure 10:**
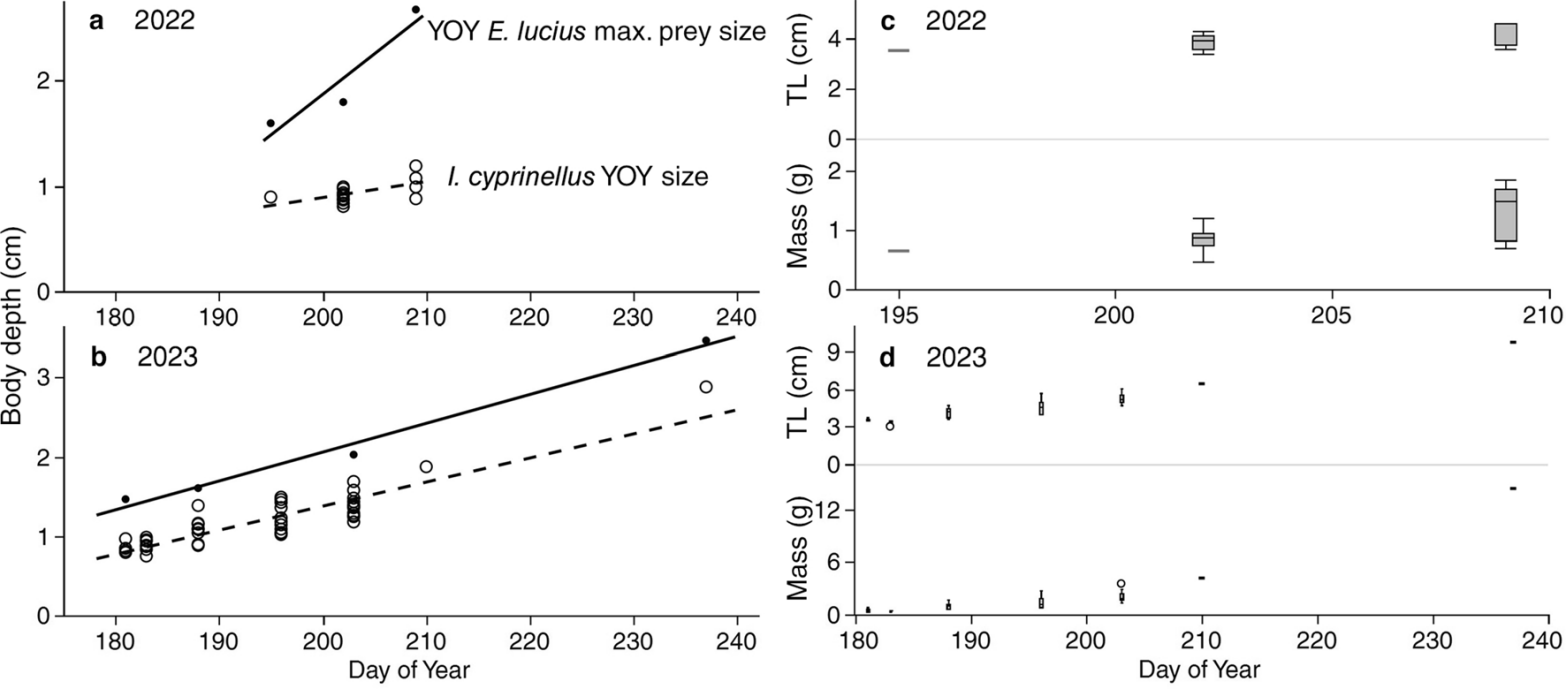
Young-of-the-year bigmouth buffalo *Ictiobus cyprinellus* size versus day of year. (**a**, **b**) RLNWR bigmouth buffalo young-of-the-year were within the gape limit of RLNWR young-of-the-year northern pike across the entire growing season in both years, as predicted from body depth^36^. (**c**, **d**) Bigmouth buffalo young-of-the year mass and total length (TL) versus day of year in 2022–2023. Box and whisker plots indicate minimum, 1st quartile, median, 3rd quartile, maximum, and outliers.

Small numbers of bigmouth buffalo young-of-the-year were captured approximately 1.5 months post-spawn, and number captured declined significantly across a 1-month span, with no captures after mid-summer regardless of gate status. Variation in CPUE was significantly explained (F_3,24_ = 3.21, *P* = 0.0411, *R*^2^ = 0.29) by day (*P* = 0.0191), but not year (*P* = 0.2051) or the interaction of day and year (*P* = 0.1891), and a post-hoc model with only day indicated a significant decline across time, slope (± SE) − 0.011 (± 0.005) (F_1,26_ = 5.72, *P* = 0.0243, *R*^2^ = 0.18). In 2022, bigmouth buffalo young-of-the-year were 0.65% of the total seining catch and they were collected on 3 of 12 sampling dates (Figs. 9, 10, Table 3). The period in which they were present spanned 14–28 July, and these bigmouth buffalo ranged from 3.4 to 4.6 cm TL, 0.46–1.83 g in mass, and 0.81–1.19 cm body depth, which were always within the gape limit of young-of-the-year northern pike present in the system across this timeframe (Fig. 10, Table 3). During the entire 2022 season the gates at the water control structure were open and fish passage was possible. All 16 young-of-the-year bigmouth buffalo collected in 2022 (across 72 total seines) were seined on the downstream side of the water control structure. In 2023, bigmouth buffalo young-of-the-year were 0.35% of the total seining catch, collected across 7 of 17 sampling dates (Figs. 9, 10, Table 4). The period in which they were present spanned from 30 June to 25 August, and these bigmouth buffalo ranged from 3.1 to 9.8 cm TL, 0.38–14.44 g in mass, and 0.75–2.88 cm body depth, which again was always within the gape limit for prey size of young-of-the-year northern pike present in the system across this timeframe (Fig. 10, Table 4). The first 49 young-of-the-year bigmouth buffalo collected in 2023 (out of 51 total collected across 85 seines) occurred across the approximately 3-week span of 30 June–22 July (Fig. 10, Table 4). The remaining two individuals were collected across four sampling dates (16 seines) from 29 July to 25 August (Fig. 10, Table 4). During 2023, the gates were closed from 12 June until 2 November, making fish passage across the barrier not possible. Indeed, all 51 bigmouth buffalo young-of-the-year collected in 2023 came from the upstream side of the water control structure, this despite 15 seine pulls on the downstream side from 30 June to 7 July, after which seining on the downstream side was discontinued until the gates re-opened (see Methods). Average rank-order abundance indicated that yellow perch *Perca flavescens*, bullheads *Ameiurus* spp., bluegill *Lepomis macrochirus*, black crappie *Pomoxis nigromaculatus*, and northern pike *Esox lucius* were the most abundant taxa (Tables 3, 4).

**Table 3:** Post-spawn seining data for young-of-the-year (YOY) fishes by date in 2022, as well as catch-per-unit effort (CPUE = number of fish/seine) for each taxon collected from Rice Lake National Wildlife Refuge. Species are in descending order of total catch. Note that the water control structure gates were open the entire 2022 season. Bigmouth buffalo (BMB) is bolded. Numbers in parentheses represent standard deviation.

	Common name	Species name	28 Jun	8 July	14 July	21 July	28 July	1 Aug	22 Aug	1 Sep	7 Sep	20 Sep	3 Oct	2 Nov	Total	CPUE
1	Yellow perch	*Perca flavescens*	100	30	20	100	35	100	20	60	60	35	35	7	602	8.36
2	Black bullhead	*Ameiurus melas*	50	100	10	15	5	100	100	60	40	25	1		506	7.03
3	Bluegill	*Lepomis macrochirus*	20	50	100	50	20	30	20	40	15	50	20	11	426	5.92
4	Black crappie	*Pomoxis nigromaculatus*		5	5	15	15	100	30	10	25	20	12	1	238	3.31
5	Brown bullhead	*A. nebulosus*	1	3		2		1	3	5	5	150	1	1	172	2.39
6	Northern pike	*Esox lucius*	19	30	15	12	10	11	12	9	11	3	6	1	139	1.93
7	Golden shiner	*Notemigonus crysoleucas*		3	25		50	10	25	15	1	1	3		133	1.85
8	Common shiner	*Luxilus cornutus*	7	3		1	10	10	5	20	2	40	20		118	1.64
9	Tadpole madtom	*Noturus gyrinus*						8	7	10	15	2	5	3	50	0.69
10	Central mudminnow	*Umbra limi*			1	1	1	7	4	3	4	4	4	1	30	0.42
11	Largemouth bass	*Micropterus salmoides*			6	2	1	5	1	2	2	2	1	3	25	0.35
**12**	**Bigmouth buffalo**	***Ictiobus cyprinellus***			**1**	**10**	**5**								**16**	**0.22**
13	Pumpkinseed	*L. gibbosus*			1		1					1	1	2	6	0.08
14	Eyetail bowfin	*Amia ocellicauda*		1	1			3							5	0.07
15	Fathead minnow	*Pimephales promelas*			5										5	0.07
16	Least darter	*Etheostoma microperca*								2				1	3	0.04
17	Blacknose dace	*Rhinichthys atratulus*			1										1	0.01
18	Rock bass	*Ambloplites rupestris*									1				1	0.01
		YOY BMB mean TL in cm (sd)			3.5	3.9 (0.3)	4.3 (0.5)									
		YOY BMB mean mass in g (sd)			0.63	0.83 (0.20)	1.29 (0.47)

**Table 4:** Post-spawn seining data for young-of-the-year (YOY) fishes by date in 2023, as well as catch-per-unit effort (CPUE = number of fish/seine) for each taxon collected from Rice Lake National Wildlife Refuge. Species are in descending order of total catch. Note that the water control structure gates were closed from 12 June until 2 November 2023, during which fish could not pass downstream of the water control structure. Bigmouth buffalo (BMB) is bolded. Number in parentheses represent standard deviation.

	Common name	Species name	30 Jun	2 July	7 July	15 July	22 July	29 July	4 Aug	13 Aug	25 Aug	26 Aug	10 Sep	15 Sep	23 Sep	30 Sep	15 Oct	28 Oct	11 Nov	Total	CPUE
1	Yellow perch	*Perca flavescens*	363	177	10,147	705	430	139	120	66	15	17	213	147	11	29	5	2	2	12,588	187.88
2	Black bullhead	*Ameiurus melas*	114	98	99	38	3	2	3			44		1					1	403	6.01
3	Black crappie	*Pomoxis nigromaculatus*			2	2	2	27	35	46	75	9	103	15	42	31	2			391	5.84
4	Bluegill	*Lepomis macrochirus*	3	5	3			4			12	11	116	78	86	28	1			347	5.18
5	Brown bullhead	*A. nebulosus*	52	24	2			3	10	9	34	10		41	72	1		3		261	3.90
6	White sucker	*Catostomus commersonii*		50		31	18	52		6	4	2		3		2				168	2.51
7	Tadpole madtom	*Noturus gyrinus*				1		33	14	19	9	3	3	2	1	2		2		89	1.33
8	Largemouth bass	*Micropterus salmoides*	1	4	5	12	7	27	5	4	4	4	6	6						85	1.27
9	Northern pike	*Esox lucius*	11	9	7	4		2	4	2	2	5		2	4	3	1	1		57	0.85
10	Golden shiner	*Notemigonus crysoleucas*	1	1	1			4	17	5	12	4	4		6					55	0.82
**11**	**Bigmouth buffalo**	***Ictiobus cyprinellus***	**4**	**8**	**9**	**12**	**16**	**1**			**1**									**51**	**0.60**
12	Pumpkinseed	*L. gibbosus*	3								1		2	2	7					15	0.22
13	Common shiner	*Luxilus cornutus*		7	1															8	0.12
14	Least darter	*Etheostoma microperca*						1		2			1	3					1	8	0.12
15	Brook silverside	*Labidesthes sicculus*											3							3	0.04
16	Creek chub	*Semotilus atromaculatus*											2		1					3	0.04
17	Rock bass	*Ambloplites rupestris*						3												3	0.04
18	Central mudminnow	*Umbra limi*	1	1																2	0.03
19	Eyetail bowfin	*Amia ocellicauda*													1					1	0.01
		YOY BMB mean TL in cm	3.6 (0.2)	3.4 (0.1)	4.2 (0.4)	4.6 (0.6)	5.3 (0.4)	6.5			9.8										
		YOY BMB mean mass in g	0.56 (0.19)	0.49 (0.06)	1.03 (0.32)	1.43 (0.63)	2.10 (0.59)	4.23			14.44										

## Discussion

Bigmouth buffalo migrate the Rice River in east-central Minnesota and enter Rice Lake National Wildlife Refuge to spawn in Rice Lake each spring, and there are sex-specific patterns in migration consistent with other long-lived, iteroparous, migratory fishes. Although bigmouth buffalo migration into Rice Lake has been anecdotally reported since at least the 1880s^29–32,37^, sex-specific migration patterns for bigmouth buffalo have not been reported anywhere across their range. We found that male bigmouth buffalo collectively arrive to the spawning grounds with females, but that males leave later. Our findings indicate that female bigmouth buffalo control the timing and duration of spawning, and that males have evolved a tendency to continue to await spawn-ready females arriving later in Rice Lake (the spawning area). Bigmouth buffalo are broadcast spawners that exhibit no parental care^4,25^, and an individual spawning act typically consists of several males aggregated around a female^4^. This general reproductive and phenological pattern has been documented in other long-lived migratory fishes such as lake sturgeon *Acipenser fulvescens*^38^, white sturgeon *A. transmontanus*^39^, green sturgeon *Acipenser medirostris*^40^, paddlefish *Polyodon spathula*^41^, and striped bass *Morone saxatilis*^42^.

The duration and timing of spawning was variable across years, with similar interannual variability in the timing and magnitude of the spring water level peak, which presents fisheries management challenges. While spawning season duration (3–4 days) for years 2021 and 2023 was not significantly different, the timing of the seasons differed by one week. On the other hand, the spawning season duration was approximately sevenfold longer in 2022 and was more than 16 days later in timing compared to 2021 (but not significantly later than 2023). The water level in mid-May 2022, which was the peak, was higher than in any of the other two years (during mid-May) by > 1 m and was due to heavy rains. The spring water level peak in 2021 occurred in mid-April before any bigmouth buffalo were observed. In 2023 spring water levels started out higher than in 2021 or 2022, but once again the peak occurred in mid-April while temperatures were near freezing and bigmouth buffalo were not yet observed in the system. Water level is positively related to bigmouth buffalo spawning success^1,4,5,43^, and potentially affects spawning duration and timing. Restricting exploitation during spawning periods is a common management practice to protect or restore threatened stocks^44^. Fisheries management strategies to protect spawning bigmouth buffalo from harvest must account for significant interannual variability in the phenology. As we have learned, during one year the spawn may conclude by early May, whereas in the very next year it may not conclude until mid-June.

The age structure of bigmouth buffalo from Rice Lake National Wildlife Refuge reveals pronounced episodic recruitment over the past 100 years and an extremely old-growth population that is one of the oldest populations of animal. Approximately 100% (99.7%) of the bigmouth buffalo in this system hatched prior to 1972, and 95.4% of the population hatched before 1960. With a median age of 79 years as of 2024 (*n* = 390), this bigmouth buffalo population is one of the oldest populations of an extant vertebrate that is definitively known. To our knowledge, the only other vertebrate populations currently known to exhibit an older or similar median age are that of the longest-lived vertebrate, the Greenland shark (*Somniosus microcephalus*)^45^, the extremely long-lived, deep-sea orange roughy *Hoplostethus atlanticus* off the coast of New Zealand^46^, and other bigmouth buffalo populations^4,7^. For Greenland shark, the median age was estimated to be 126 years as of 2012 (*n* = 28)^45^, whereas for orange roughy the median age was approximately 50 years as of 2018 (*n* ~ 550)^46^. Several bigmouth buffalo populations exhibit age distributions skewed towards older individuals, likely due to their longevity, reproductive ecology, and use of spawning habitats that experience significant interannual variation in water conditions that affect recruitment^4–7^. For example, bigmouth buffalo from the Pelican River watershed of northwestern Minnesota (near the city of Detroit Lakes) were found to have a median age of 88 years as of 2019 (*n* = 224)^6^, while a population from Buffalo Pound Lake (near Moose Jaw, Saskatchewan) were found to have a median age of 79 years as of 2023 (*n* = 52)^4^, and a population from Apache Lake, Arizona revealed a median age of 98 years as of 2021 (*n* = 18)^7^. Bigmouth buffalo have repeatedly shown a tendency to harbor some of the oldest populations of animal currently known.

Assuming natal site fidelity (which evidence suggests is prevalent across various migratory catostomids^47–50^), we estimate more than six decades of no substantial recruitment of bigmouth buffalo at RLNWR even as the population spawns annually. Temporal gaps of decades between successful recruitment of bigmouth buffalo have been observed elsewhere^4–7^, including cases where bigmouth buffalo may skip spawning for some or many years^4^. However, we confirmed spawning occurred in Rice Lake in each of the three years of this study. Furthermore, long-time observers, employees of RLNWR and Minnesota Department of Natural Resources (MNDNR), buffalofish spearfishers, bowfishers, and anglers have collectively noted the bigmouth buffalo spawning migration at the refuge every spring since at least 1978, as well as numerous accounts of observed spawning activity in Rice Lake itself (J. Francis, USFWS RLNWR, 1978–2005 pers. obs.; S. Seybold Aitkin County Soil and Water Conservation District (ACSWCD), 1986–2005, 2020–2023, pers. obs.; W. Ford, USFWS RLNWR 2006–2023; observations from various bowfishers, spearfishers, anglers—personal communication with A. Lackmann 2021–2023;^31^). Moreover, the bigmouth buffalo Rice Lake spawning migration has been documented as an annual event in old reports during the 1940s and in accounts dating to the 1880s^29,30,32^. We verified spawning occurred 2021–2023 and conclude that these observations were not exceptional. Therefore, spawning has likely occurred annually in Rice Lake since 1880 or earlier and factors other than failure to spawn are responsible for the lack of evidence of recruitment in the population before 1926 and since 1958.

After year 1958 all remaining recruited individuals (4.6% of total) are statistical outliers. Although such a long period (> 60 years) with negligible recruitment is possibly unparalleled across vertebrates, this may be typical for bigmouth buffalo considering their longevity (> 125 years), reproductive ecology, and slow life history strategy^4,6^. Nonetheless, there is evidence that recruitment declined significantly in the post-water control era (after 1952), using either 1926 or 1895 as starting points for the preceding era. However, the natural age ceiling of bigmouth buffalo lifespan is not well understood^7,28^ in part because they have repeatedly shown a tendency for episodic recruitment^4–7^. It is unclear, for example, how long the recruitment gap was pre-1926 at RLNWR, but it is conceivable that it could have been 30 years or more.

The lakeside water control structure does affect the movement of bigmouth buffalo in this system. For example, during some springs all gates have been closed or nearly closed, which creates a barrier to migration. During one spring in the late 1990s when the gates of the water control were closed so far that bigmouth could not pass, large aggregations of bigmouth buffalo (pre-spawn females were definitively noted) were observed waiting beneath the water control structure for nearly 40 days (1 May–7 June; S. Seybold pers. obs.). During one extremely high-water year in May of 1986, bigmouth buffalo were observed spawning in freshly flooded terrestrial vegetation along the Rice River within a few hundred meters downstream of the lakeside water control structure (S. Seybold pers. obs.). During this time the gates may have been functionally closed as well.

During years that the gates remain open to the spawning migration of bigmouth buffalo movement appears to be affected. A consistent observation across all years of this study (2021–2023) and in previous years, was that bigmouth buffalo aggregate just downstream of the open gates (e.g., Fig. 1c), before passing through. We observed individuals with unique markings swimming up to the gates, going part-way in but then reversing, and repeating this process numerous times before eventually passing completely through the gates. We hypothesize that the concrete walls and narrow channels within each of the gates amplify sound as well as increase the stream velocity at this point, and that this affects navigation. Any substantial vibration when bigmouth buffalo are congregated near the concrete gates causes the aggregation of bigmouth buffalo to retreat downstream for a period of about 5 min. Footsteps on the metal grates, angler activity, car doors slamming, vehicles approaching, planes flying overhead, or even trees falling ~ 80 m away during high winds, were all situations observed repeatedly to cause the bigmouth buffalo to startle and retreat when at the water control structure. Nonetheless, bigmouth buffalo are a skittish species by nature (A. Lackmann, pers. obs. 2011–2023).

The year class distribution of bigmouth buffalo from Rice Lake National Wildlife Refuge reveals a successful recruitment period that largely occurred in the early 1940s and early 1950s. Although there is no long-term history (50–100 years ago) of quantitative water level data for Rice Lake, there are mentions of drought or high water years in RLNWR-specific reports. Johnson^30^ recalls the drought years of 1930 and 1932–1934, with specific mention that in 1930 the water levels were so low that the “…whole lake looked like an enormous wheat field.” Although it is not clear what year 1931 was like, we found evidence of 12 individuals from the 1931 year class, which is the year following the memorable drought of 1930. Johnson^30^ also indicates a period between 1940 and 1944 that had such “extreme high water” that it exponentially reduced muskrat numbers due to ice heaves in the winter. When describing this period of the early 1940s on Rice Lake, he also mentioned that Mud Island changed position “…during the high water of the past few years…” We found that 1942 is the most abundant year class in the bigmouth buffalo population as of the 2020s, which is a year class that appears to coincide with a time of extremely high water as mentioned by Johnson^30^ long ago.

Ustipak^32^ also reports on highlights throughout the history of Rice Lake National Wildlife Refuge and mentions that during years 1952 and 1953 there was such extremely high water that it hindered construction of the Rice River Pool water control structure. The second-most abundant year class in the sample was from 1950, which is near this time. The magnitude and timing of the spring peak in water level, as well as the water level recession rate post-peak, are all environmental factors known to be associated with the recruitment success of bigmouth buffalo^1,4,5,43^. The single year class represented after 1971 (2012; *n* = 1 individual), was the year during which there was an extreme flood in the latter half of June that resulted in the spring peak water level (although not in May) at the 100th percentile for available data (26 years across the span of 1990–2023).

The RLNWR system could be studied to determine the maximum spawning frequency of individual bigmouth buffalo. Previous reports on the species found evidence that not all mature females can spawn each year^1,43^. It is possible that bigmouth buffalo may take years to regenerate gonadal tissue post-spawn, much like the long-lived lake sturgeon^38^. However, since spawning occurs in the Rice Lake system annually it would suggest that either (1) individuals in this system are capable of spawning in successive years or (2) individuals are staggered across years (like in lake sturgeon)^51^. It is unknown where bigmouth buffalo within the Rice River system are migrating from, but this could be determined through mark-recapture or tagging efforts. Although a valid migratory channel, the Rice River itself is not suitable residence habitat for bigmouth buffalo because bigmouth buffalo are open water planktivores^1,52^. Rice River averages 21 m wide and 0.76 m deep and is often in a state of drought throughout the summer^29^. Rice Lake is also unsuitable residence habitat for bigmouth buffalo because it is very shallow (< 2 m throughout, commonly < 1 m), subject to winterkill, and approximately 0% of its habitat is pelagic due to the vast stands of wild rice and other vegetation throughout the lake^29^. To determine where bigmouth buffalo are migrating from, the unique phenotypic markings that these bigmouth buffalo have naturally accumulated over their long lives (black or orange spots, white-edged fins in some cases) could be used to support mark recapture^6,7^ or tagging efforts. We hypothesized that the presence of white markings on old-age bigmouth buffalo may be associated with the absence of orange spot pigmentation on old-age (> 50 years) bigmouth buffalo. However, evidence indicated this was not the case. It is possible that these white markings are evidence of piebaldism in mature individuals, as has been found in other fishes^53^, and genetic studies are needed.

The gonadal investment of large bigmouth buffalo females is disproportionately more than their smaller female counterparts, which has implications for developing size-based management. This raises yet another concern about unlimited, indiscriminate, and unmanaged exploitation of bigmouth buffalo that occurs in Minnesota and elsewhere. If larger females contribute more to reproductive output, then at the individual level they may be more valuable to the reproductive success of the population, the principal assumption underlying size-restricted or size-slotted harvest regulations^54^. A positive association between individual body size and reproductive success has been found for lake sturgeon^55^, and future genetic studies at RLNWR could test this hypothesis for bigmouth buffalo. In order to estimate contributions of female size classes to population-level offspring production for size-based or size-slotted harvest regulations in bigmouth buffalo, we identify three questions for inquiry related to the increase in GSI with body size: (1) how does egg size vary with female body size, (2) how does egg quality vary with female size, and (3) how do size classes vary in magnitude in the population. Sex-specific energy expenditure of bigmouth buffalo across the spawn is another line of investigation worth evaluating. For other long-lived, protected, migratory, iteroparous freshwater fishes such as paddlefish and sturgeon, it has been hypothesized that females expend more energy across a spawn^38,41,51^. Interestingly, the largest individual collected was a post-spawn female that was 18.20 kg and 80 years old. Based on GSI data of the population, it is likely that this individual had 6–7 kg of gonad when it was pre-spawn and was between 24 and 25 kg in total body mass (52.9–55.1 pounds). This individual suggests that bigmouth buffalo as large as 22.68 kg (50 pounds) inhabit the system, an observation that was estimated by Johnson^30^ 80 years ago.

The standard deviation of bigmouth buffalo adult asymptotic size in TL, within each sex, exceeds their estimated annual growth rate (0.1 cm) by more than an order of magnitude. Since 99.7% of bigmouth buffalo were 51 years or older and are part of a single migrating population, it provides an opportunity to investigate variation in asymptotic body size, and potential growth at approximately asymptotic ages. We found that regardless of sex, bigmouth buffalo appear to grow in TL at a rate of 0.1 cm per year across ages spanning 51–96 years old. This growth rate is trivial in comparison to the variability in size at any given age within this range. For example, the most abundant age class was 80 years (female *n* = 19, male *n* = 24). Females within this age class exhibit a standard deviation of 7.0 cm in TL (TL ranged from 81.2 to 104.0 cm), whereas males showed a standard deviation of 3.8 cm (TL ranged from 68.6 to 84.0 cm). Furthermore, the largest asymptotically mature individuals were not the oldest, and the smallest asymptotically mature individuals were not the youngest. These observations, amidst the size variability at any given age, contradict the assumption that significantly larger fish are significantly older^45^. Nonetheless, bigmouth buffalo of asymptotically mature ages increased on average by 1 mm in TL per year, which is marginal evidence that appears to contradict asymptotic growth models commonly used for fishes (e.g.,^56^). More study into the mechanisms regulating growth of bigmouth buffalo is needed.

Overall, it is perhaps most remarkable that bigmouth buffalo of Rice Lake National Wildlife Refuge have failed to recruit so often during the past 66 years, but this remarkable fact is supported by numerous lines of evidence. First, the age structure of the population indicates that substantial recruitment has not occurred since the late 1950s, and that absolute recruitment failure has occurred for 98% of years since 1971, even as bigmouth buffalo have been spawning in this system annually. Second, the one successful year class since 1971 is from 2012, which is a year class that represents < 0.3% of the bigmouth buffalo stock. Third, extensive fall and winter “fish rescue” netting efforts by MNDNR since the 1970s have not uncovered any juvenile or adult bigmouth buffalo in Rice Lake (or at the lakeside water control structure) despite the consistent presence of spawning bigmouth buffalo each spring and the presence of northern pike, yellow perch, white sucker, various centrarchids, and bullheads throughout the entire year (MNDNR, personal communication with A. Lackmann, 2024)^31^. Fourth, during many of these years the Rice Lake water control gates were closed during summer, which would prevent bigmouth buffalo young from migrating out of the system (MNDNR, personal communication with A. Lackmann, 2024)^31^, yet still none were collected. Fifth, in 2019 the MNDNR sampled RLNWR for its first (and so far only) MNDNR standard fish survey^31^, and is the only record of bigmouth buffalo collected from RLNWR by the MNDNR since the 1960s (MNDNR data received upon request, 2024). This 2019 sampling occurred during a 3-day span in May, standardized netting procedures were employed designed to collect all fish sizes from yellow perch to bigmouth buffalo, and 47 bigmouth buffalo were collected ranging from 67.4 to 90.5 cm TL, mean = 77.3, median = 75.7 cm TL^31^. This 2019 size structure is consistent with an asymptotically mature population of bigmouth buffalo, and consistent with the size-structure of bigmouth buffalo collected in this study (2021–2023). Sixth, extensive seining across the growing seasons of 2022 and 2023 post-spawn revealed that bigmouth buffalo young-of-the-year were present in very low numbers initially, but that they were no longer evident by mid-to-late summer no matter if the water control gates were open or closed (not allowing fish passage out of the system). Seventh, the overall scarcity of bigmouth buffalo young in some systems following spawn is not uncommon. For example, extensive seining across the entire growing season in several other systems and years has resulted in few (if any) young-of-the-year bigmouth buffalo, even though spawning was observed in those very years^1,4,43^. Eighth, anecdotal reports from long-time observers (1978–2023) consistently indicate that the adult population of bigmouth buffalo has declined significantly over this timeframe (J. Francis, USFWS RLNWR, pers. obs. 1978–2005; S. Seybold, ACSWCD, personal observations 1986–2023). Ninth, and perhaps most importantly, the extreme lifespan and other long-lived traits of bigmouth buffalo are a unique adaptive response to the myriad complex challenges that shallow freshwater ecosystems create, traits that allow the species to bridge long gaps when unfavorable recruitment conditions occur^4^.

In aggregate, it is therefore imperative that the current cohort of adults that is the demographic store of this bigmouth buffalo population is protected until the next favorable recruitment period arises, while study of the proximate factors that determine recruitment success are investigated. As can be seen at RLNWR in east-central Minnesota and in the Pelican River watershed of northwestern Minnesota^6^, this species is at serious risk of extirpation across parts of its range where recruitment has not occurred for the past 40–50 years and no fisheries management protections continue amidst rising exploitative fisheries. We strongly recommend that conservation plans be implemented immediately for bigmouth buffalo in Minnesota and elsewhere, and that their conservation status be reassessed and updated by the IUCN and NatureServe.

We identify three hypotheses for why bigmouth buffalo recruitment has persistently failed at Rice Lake since 1958. First, abiotic conditions may not be favorable for survival of young-of-the-year. Historical data on water temperatures, water level fluctuations, and water chemistry (e.g., dissolved O_2_ levels) were insufficient to compare with the year class data from bigmouth buffalo captured at Rice Lake to potentially assess this hypothesis. However, growth rates of young-of-the-year captured in 2023 at Rice Lake were comparable to rates observed in Canada during periods with successful recruitment^1^, which is inconsistent with a view of water conditions limiting growth as a mechanism underlying recruitment failure. Second, outmigration of young-of-the-year bigmouth buffalo is restricted at Rice Lake, which may prevent young-of-the-year from moving to juvenile habitat appropriate for survival. In 2023, the lakeside control structure was closed, which prevented movement of fish out of Rice Lake until November, and all young-of-the-year captures occurred immediately upstream of the lakeside of the water control structure, whereas in 2022 when the control gates were open all bigmouth buffalo young-of-the-year were captured immediately downstream of the water control structure. Adult bigmouth buffalo leave the lake immediately after spawning, but there remains a gap in our knowledge of the age at which their young-of-the-year leave the shallow, vegetated habitat used for spawning. Third, piscivory of bigmouth buffalo young-of-the-year by other fish in Rice Lake may prevent recruitment. For example, the size of bigmouth buffalo young-of-the-year never exceeded the gape limit of young-of-the-year northern pike present in Rice Lake (Fig. 10), which is consistent with a hypothesis that survival is top-down restricted. Note these hypotheses need not be mutually exclusive. For instance, operation of the lakeside control structure could have altered water level dynamics, water temperatures, or dissolved O_2_ levels from pre-control conditions to a regime that now enables young-of-the-year piscivorous fish to survive in abundance and prevent bigmouth buffalo young-of-the-year survival. This scenario would be consistent with all the hypotheses. Studies focused on young-of-the-year recruitment are a necessary next step during the conservation of this species.

Overall, we hypothesize that the primary proximate reason for consistent recruitment failure in this system is that bigmouth buffalo offspring completely succumb to predation pressure (insufficient predator swamping) during almost all years. For example, northern pike are a voracious native predator that spawns in the same Rice Lake habitat approximately one month prior to bigmouth buffalo, and this results in a substantial young-of-the-year size differential for which even young-of the-year northern pike are capable of consuming young-of-the-year bigmouth buffalo across the entire season (Fig. 10). In addition, RLNWR is known for its excellent northern pike fishery and adult pike inhabit the system year-round^29^. Furthermore, there are numerous other aquatic predators that appear to key in on the nutrient pulse provided by spawning bigmouth buffalo, a large resource nutrient swing that is possibly entrenched across evolutionary time and could parallel that of the nutrient swings provided by migrating salmonids^57^. These other predators on bigmouth buffalo include water mites (Hydrachnidia), *Hydra* spp., Odonata, Dytiscidae, bluegills, black crappie, yellow perch, bullheads, largemouth bass, and many other taxa that either prey on the eggs during embryogenesis, larvae post-hatch, or later stages of bigmouth buffalo development (A. Lackmann pers. obs. 2011–2023). The predator–prey dynamics within the bigmouth buffalo post-spawn ecosystem is an area of research in specific need of study. In addition, there are two primary factors that are known to impact bigmouth buffalo pre-spawn in this system: water control and exploitation. The impact of water control and human exploitation on adult bigmouth buffalo is confounded because both began around the same time, that is the narrowing of the river that was created for water control also created the opportunity for increased angler exploitation (J. Francis, USFWS RLNWR, 1978–2005 pers. obs.; S. Seybold ACSWCD, 1986–2023, pers. obs.). In addition, for the past 70 years all that has been required for anglers to target spawn-ready migrating bigmouth buffalo, with no limit, is a fishing license^18^ at this location within Rice Lake National Wildlife Refuge. It is unknown how many adults have been removed across this timeframe and what cumulative effect this may be having on the spawning population as decades have passed with no recruitment. Future study on the various factors that may influence bigmouth buffalo recruitment success across diverse systems is needed.

This work demonstrates the power of putting forth effort to study something neglected. For decades bigmouth buffalo have been cast aside as inconsequential, even as they have become increasingly exploited by anglers. Little did we know that some of the bigmouth buffalo still migrating and spawning in Rice Lake National Wildlife Refuge pre-date the refuge establishment itself (1935), let alone that this system belongs to one of the oldest extant vertebrate populations known. Indeed, this is a population of bigmouth buffalo with potential to provide profound insight to the study of ecology, while also inspiring awe in those who can witness their age-old migration. Here again, bigmouth buffalo serve as a prime example of the discoveries overlooked and the management dilemmas that can arise as a consequence of the ecological neglect of underappreciated species. Conservation concerns must be addressed for this iconic species, and it all starts with implementing basic principles of fisheries management. The increasing and unlimited take of a wild, extremely long-lived, declining freshwater fish with no active management is antithetical to resource conservation and is contrary to the establishment of wildlife refugia. The bigmouth buffalo is testament to the complexity of freshwater ecosystems and the many unsolved mysteries of the natural world. For all these reasons this species is worth preserving.

## Data Availability

The data that support the findings of this study are available from the corresponding author upon reasonable request.
